# An Auxiliary Logic on Trees: on the Tower-Hardness of Logics Featuring Reachability and Submodel Reasoning

**DOI:** 10.1007/978-3-030-45231-5_24

**Published:** 2020-04-17

**Authors:** Alessio Mansutti

**Affiliations:** 8 grid.460789.4 0000 0004 4910 6535 ENS/CNRS, Université Paris-Saclay, Cachan, France; 9 grid.5718.b 0000 0001 2187 5445 University of Duisburg-Essen, Duisburg, Germany; grid.460789.4 0000 0004 4910 6535 LSV, CNRS, ENS Paris-Saclay, Université Paris-Saclay, Cachan, France

## Abstract

We describe a set of simple features that are sufficient in order to make the satisfiability problem of logics interpreted on trees Tower-hard. We exhibit these features through an *Auxiliary Logic on Trees* (ALT), a modal logic that essentially deals with reachability of a fixed node inside a forest and features modalities from sabotage modal logic to reason on submodels. After showing that ALT admits a Tower-complete satisfiability problem, we prove that this logic is captured by four other logics that were independently found to be Tower-complete: two-variables separation logic, quantified computation tree logic, modal logic of heaps and modal separation logic. As a by-product of establishing these connections, we discover strict fragments of these logics that are still non-elementary.

## 1 Introduction

In mathematical logic there is a well-known trade-off between expressive power and complexity, where weaker languages cannot capture interesting properties of complex systems, whereas finding solutions of a given problem is infeasible for richer languages. For instance, many verification tasks, such as reachability and homomorphisms queries, happen to be expressible in monadic second-order logic (MSO) [15]. This logic is however not usable in practice, as its satisfiability problem SAT(MSO) is undecidable in general and was famously proved by Rabin [36] to be decidable but non-elementary when the logic is interpreted on trees or on one unary function. A more recent analysis that uses the hierarchy of non-elementary ranking functions [38] classifies SAT(MSO) on these two structures as TOWER-complete, i.e. complete for the class of problems of time complexity bounded by a tower of exponentials, whose height is an elementary function of the input.

In order to bypass these problems, a general approach is to design restrictions of MSO that can solve complex reasoning tasks while being more appealing complexity-wise. An example of this is given by the framework of temporal logics, formalisms that describe the evolution of reactive systems [24]. Among the various temporal logics, from the classical linear temporal logic (LTL) [39] and computation tree logic (CTL) [13], as well as their fragments [2,33], to the more recently developed interval temporal logics [7,8], the main common feature of this framework is perhaps the ability to check whether the system can evolve to a certain configuration, i.e. a *reachability* query. In this context, we recall the landmark result on the satisfiability of CTL, shown EXPTIME-complete by Fisher and Ladner [23]. Another possibility to deal with the complexity of MSO is to restrict the second-order quantifications to specific *submodels*. This is the idea behind ambient logic [16], separation logic [37] and more generally bunched logics [35]

and graphs logics [1]. These logics provide primitives for reasoning about resource composition, mainly by adding a *spatial conjunction* $\varphi * \psi$ which requires to split a model into two disjoint pieces, one satisfying $\varphi$ and the other satisfying $\psi$. Similar ideas are developed in sabotage modal logics, where the formula $\blacklozenge\,\varphi$, headed by the *sabotage* modality $\blacklozenge$, states that $\varphi$ must hold in a graph obtained by removing one edge from the current model [4,21]. Within these logics, we highlight the quantifier-free fragment of separation logic restricted to the $*$ operator, denoted here with SL($*$) and whose satisfiability problem is proved to be PSPACE-complete in [12].

Once a framework provides a solid foundation for reasoning tasks, a natural step is to extend its expressiveness while keeping its complexity in check. Sometimes the additional capabilities do not change the complexity of the logic, as for example SL($*$) extended with reachability predicates, whose satisfiability problem is still PSPACE-complete [20]. However, it often happens that the new features make the problem jump to higher complexity classes and, sometimes, reach MSO. We pinpoint two instances of this:

- SL($*$) enriched with first-order quantifiers, albeit less expressive than MSO interpreted on one unary function, has a TOWER-complete satisfiability problem [9].
- CTL enriched with propositional quantifiers has an undecidable satisfiability problem on general models. On trees (i.e. QCTL$^T$), the problem is TOWER-complete [28].

Consequently, it is natural to ask ourselves why the additional features made the problem harder. Answering this question requires to study the interplays between the various operators of the logic, searching for a sufficient set of conditions explaining its complexity.

**Our motivation.** Second-order features often lead to logics with TOWER-hard satisfiability problems, as illustrated above for first-order SL($*$) and QCTL$^T$. A good amount of research has been done independently on these logics [5,9,17,28], culminating with the TOWER-hardness of SL($*$) with two quantified variables [17] and the TOWER-hardness of QCTL$^T$ with just one temporal operator between *exists-finally* EF and *exists-next* EX [5] (see Section 4 for the definitions). Connections between these two formalisms have not been explicitly developed so far, perhaps because of the quite different logics: QCTL$^T$ is built on top of propositional calculus and it is interpreted on infinite trees, whereas SL($*$) does not feature propositional symbols and it is essentially interpreted on finite structures. Nevertheless, we argue that these and other logics are related not only as they are fragments of MSO, but also as they share a form of reachability and an ability of reasoning on submodels which is sufficient to obtain TOWER-hard logics.

**Our contribution.** We explicit these common features that lead to TOWER-hard logics by relying on an *Auxiliary Logic on Trees* (ALT), introduced in Section 2. ALT reasons about reachability of a fixed *target node* inside a finite forest and features modalities from sabotage logics to reason on submodels. Here, reachability should be understood as the ability to reach the target node in at least one step, starting from a "current" node which can be updated thanks to the existential modality *somewhere* $\langle \mathrm{U} \rangle$ [26]. In Section 3, we take a look at the expressive power of ALT and show that SAT(ALT) is TOWER-hard. In Section 4, we then display how ALT is captured by first-order SL($*$) and QCTL$^T$, as well as modal logic of heaps (MLH) and modal separation logics (MSL), two other logics introduced in [17] and [18], respectively. In this context, beside exposing that all these logics are TOWER-hard because of the way they reason about reachability and submodels, we discover interesting sublogics that are still TOWER-complete:

- $\mathsf{QCTL}^T$ restricted to $\mathsf{E}(\varphi \mathbin{\mathsf{U}} \psi)$ modalities, where $\varphi, \psi$ are Boolean combinations of atomic propositions, or to the $\mathsf{EF}$ modality, which can be nested at most once.
- the common fragment of MLH and MSL having Boolean connectives and the modalities $\Diamond$, $\langle \mathrm{U} \rangle$ and $*$. Notice that this logic does not have propositional symbols.

## 2 The definition of an Auxiliary Logic on Trees

We introduce an Auxiliary Logic on Trees (ALT). Its formulae are from the grammar:

$$\varphi := \top \mid \varphi \wedge \varphi \mid \neg\varphi \mid \mathtt{T} \mid \mathtt{G} \mid \langle \mathrm{U} \rangle\, \varphi \mid \blacklozenge \varphi \mid \blacklozenge^{*} \varphi$$

As we will soon clarify, the symbol $\langle \mathrm{U} \rangle$ is borrowed from Goranko and Passy paper on modal logic with universal modality [26]. Similarly, readers who are familiar with sabotage modal logics will recognise in $\blacklozenge$ the sabotage modality [4], and in $\blacklozenge^{*}$ its Kleene closure (i.e. $\blacklozenge$ applied an arbitrary number of times). These two operators modify the model during the evaluation of a formula, making ALT a *relation-changing* modal logic (following the terminology used in [3]). However, contrary to most modal logics, ALT does not feature classical propositional symbols. Instead, this logic only features two interpreted atomic propositions $\mathtt{T}$ and $\mathtt{G}$. Roughly speaking, $\mathtt{T}$ stands for "the target node is reachable" whereas $\mathtt{G}$ stands for "the target node is not reachable". The formal definitions will be given soon in order to clarify these two sentences.

Let $\mathcal{N}$ be countably infinite set of *nodes*. A *(finite) forest* $\mathcal{F} : \mathcal{N} \to_{\mathtt{fin}} \mathcal{N}$ is a partial function (encoding the standard parent relation) that

- has a finite domain of definition, i.e. $\mathrm{dom}(\mathcal{F}) \stackrel{\mathsf{def}}{=} \{\mathsf{n} \in \mathcal{N} \mid \mathcal{F}(\mathsf{n}) \text{ is defined}\}$ is finite;
- is acyclic, i.e. for every $\mathsf{n} \in \mathrm{dom}(\mathcal{F})$ and $\delta \geq 1$, $\mathcal{F}^{\delta}(\mathsf{n}) \neq \mathsf{n}$.

Here, $\mathcal{F}^{\delta}$ denotes $\delta \geq 0$ *functional composition(s)* of $\mathcal{F}$. Albeit non-standard, our definition of finite forests over an infinite set of nodes simplifies the forthcoming definitions. Besides, in Section 3.2 we show how restricting $\mathcal{N}$ to a finite set does not change the expressive power nor the complexity of ALT.

We denote the image of $\mathcal{F}$ as $\mathrm{ran}(\mathcal{F}) \stackrel{\mathsf{def}}{=} \{\mathsf{n}' \mid \mathcal{F}(\mathsf{n}) = \mathsf{n}' \text{ for some } \mathsf{n} \in \mathrm{dom}(\mathcal{F})\}$. Given a finite set $X$, we denote with $|X|$ its cardinality. Let $\mathsf{n}, \mathsf{n}'$ be two nodes. As usual, $\mathsf{n}$ is a *$\mathcal{F}$-descendant* of $\mathsf{n}'$ (alternatively, $\mathsf{n}'$ is an *$\mathcal{F}$-ancestor* of $\mathsf{n}$) whenever $\mathcal{F}^{\delta}(\mathsf{n}) = \mathsf{n}'$ for some $\delta \geq 1$. In this case, if $\delta = 1$ then $\mathsf{n}$ is a *$\mathcal{F}$-child* of $\mathsf{n}'$ (alternatively, $\mathsf{n}'$ is the *$\mathcal{F}$-parent* of $\mathsf{n}$). We drop the prefix $\mathcal{F}$- from these terms when it is clear from the context. Given two forests $\mathcal{F}, \mathcal{F}'$, we say that $\mathcal{F}'$ is a *subforest* of $\mathcal{F}$, written $\mathcal{F}' \sqsubseteq \mathcal{F}$, whenever $\mathcal{F}(\mathsf{n}) = \mathcal{F}'(\mathsf{n})$ for every $\mathsf{n} \in \mathrm{dom}(\mathcal{F}')$. Figure 1 intuitively represents two forests (every "$\square$" represents a node), the one on the left being a subforest of the one on the right.

ALT is interpreted on *pointed forests* $(\mathcal{F}, \mathsf{t}, \mathsf{n})$, where $\mathcal{F}$ is a forest and $\mathsf{t}, \mathsf{n} \in \mathcal{N}$ are respectively called the *target node* and the *current evaluation node*. The satisfaction relation $\models$ is defined (throughout the paper, we omit standard clauses for $\top, \wedge, \neg$) as:

$(\mathcal{F}, \mathsf{t}, \mathsf{n}) \models \mathtt{T} \quad \stackrel{\mathsf{def}}{\Leftrightarrow}$ $\mathsf{n}$ is a $\mathcal{F}$-descendant of $\mathsf{t}$.
$(\mathcal{F}, \mathsf{t}, \mathsf{n}) \models \mathtt{G} \quad \stackrel{\mathsf{def}}{\Leftrightarrow}$ $\mathsf{n} \in \mathrm{dom}(\mathcal{F})$ and $(\mathcal{F}, \mathsf{t}, \mathsf{n}) \not\models \mathtt{T}$.
$(\mathcal{F}, \mathsf{t}, \mathsf{n}) \models \langle \mathrm{U} \rangle\, \varphi \quad \stackrel{\mathsf{def}}{\Leftrightarrow}$ there is $\mathsf{n}' \in \mathcal{N}$ s.t. $(\mathcal{F}, \mathsf{t}, \mathsf{n}') \models \varphi$.
$(\mathcal{F}, \mathsf{t}, \mathsf{n}) \models \blacklozenge \varphi \quad \stackrel{\mathsf{def}}{\Leftrightarrow}$ there is $\mathcal{F}'$ s.t. $\mathcal{F}' \sqsubseteq \mathcal{F}$, $|\mathrm{dom}(\mathcal{F}')| + 1 = |\mathrm{dom}(\mathcal{F})|$, $(\mathcal{F}', \mathsf{t}, \mathsf{n}) \models \varphi$.
$(\mathcal{F}, \mathsf{t}, \mathsf{n}) \models \blacklozenge^{*} \varphi \quad \stackrel{\mathsf{def}}{\Leftrightarrow}$ there is $\mathcal{F}'$ s.t. $\mathcal{F}' \sqsubseteq \mathcal{F}$ and $(\mathcal{F}', \mathsf{t}, \mathsf{n}) \models \varphi$.

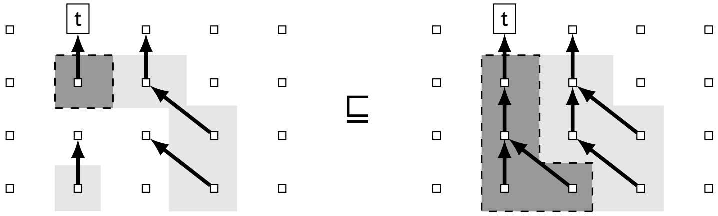

**Fig. 1.** Subforest relation

We denote with ⊥ the contradiction ¬T. The standard connectives ∨ and ⇒ are defined as usual. The semantics of T and G is pretty straightforward. As a visual aid, the nodes in Figure 1 satisfying T are the ones in the dark grey area, whereas the ones in the light grey area satisfy G. As stated before, the semantics given to $\langle U \rangle\, \varphi$ is the one of the existential modality *somewhere* [26], stating that there is a way to change the current evaluation node so that $\varphi$ becomes true. Its dual operator $[U]\, \varphi \stackrel{\text{def}}{=} \neg \langle U \rangle\, \neg\varphi$ is the universal modality *everywhere*. The semantics given to $\blacklozenge\, \varphi$ is the one of the *sabotage* modality from [4], which requires to find one edge of the forest that, when removed, makes the model satisfy $\varphi$. Lastly, the $\blacklozenge^*$ modality, here called *repeated sabotage* operator, can be seen as the operator obtained by applying $\blacklozenge$ an arbitrary number of times. Indeed, by inductively defining $\blacklozenge^k\, \varphi$ ($k \in \mathbb{N}$) as the formula $\varphi$ for $k = 0$ and otherwise ($k \geq 1$) as $\blacklozenge \blacklozenge^{k-1}\, \varphi$, it is easy to see that $(\mathcal{F}, \mathsf{t}, \mathsf{n}) \models \blacklozenge^* \varphi$ is equivalent to $\exists k \in \mathbb{N}.\ (\mathcal{F}, \mathsf{t}, \mathsf{n}) \models \blacklozenge^k\, \varphi$.

Given a pointed forest $(\mathcal{F}, \mathsf{t}, \mathsf{n})$, we denote with $\mathcal{F}(\mathtt{G})_\mathsf{t}$ the set of its *garbage nodes*: the set of elements in dom($\mathcal{F}$) that are not descendants of t, i.e. $\mathcal{F}(\mathtt{G})_\mathsf{t} \stackrel{\text{def}}{=} \{\mathsf{n} \in \mathrm{dom}(\mathcal{F}) \mid \forall\delta \geq 1,\ \mathcal{F}^\delta(\mathsf{n}) \neq \mathsf{t}\}$. Then, $\mathcal{F}(\mathtt{G})_\mathsf{t}$ is equivalent to $\{\mathsf{n} \in \mathcal{N} \mid (\mathcal{F}, \mathsf{t}, \mathsf{n}) \models \mathtt{G}\}$. We omit the subscript t from $\mathcal{F}(\mathtt{G})_\mathsf{t}$ when it is clear from the context. We augment the standard precedence rules of propositional logic so that the modalities ⟨U⟩, $\blacklozenge$ and $\blacklozenge^*$ have the same precedence as ¬. For instance, the formula $\langle U \rangle\, \mathtt{T} \wedge \mathtt{G}$ should be read as $(\langle U \rangle\, \mathtt{T}) \wedge \mathtt{G}$.

**Satisfiability problem.** As usual, given a logic $\mathfrak{L}$ and one of its interpretations ⊨ on a class of structures $\mathfrak{C}$, the satisfiability problem of $\mathfrak{L}$, denoted with SAT($\mathfrak{L}$) when the interpretation is clear from the context, takes as input a formula $\varphi$ of $\mathfrak{L}$ and asks whether there is a structure $\mathfrak{M} \in \mathfrak{C}$ such that $\mathfrak{M} \models \varphi$. If the answer is positive, then $\varphi$ is *satisfiable*.

**Where does ALT come from?** A preliminary definition of ALT was introduced in [31] to reason on the complexity of separation logic. As such, in [31] ALT features the separating conjunction $\varphi * \psi$ from separation logic, stating that the forest can be partitioned into two disjoint subforests, one satisfying $\varphi$ and one satisfying $\psi$. This binary operator generalises both $\blacklozenge$ and $\blacklozenge^*$ operators (we show how in Section 4). Hence, the TOWER-hardness of the satisfiability problem for the logic defined here cannot be inherited from [31] and must be proved (Section 3). Unfortunately, the proof does not give any indication on whether or not the two versions of ALT have the same expressive power. What is clear is that the two logics analyse the model in a different way: the $*$ operator is able to reason on the model in a "concurrent" way, whereas $\blacklozenge$ and $\blacklozenge^*$ do it in a "sequential" one. Let us draw an example of this. Let $(\mathcal{F}, \mathsf{t}, \mathsf{n})$ be a pointed forest. We aim at defining a formula $\#\mathtt{ch}_{\mathtt{trg}} \geq 2$ stating that the target node t has at least two children. First, we define $\#\mathtt{ch}_{\mathtt{trg}} \geq 1$ (the formula for just one child) as $\langle U \rangle(\mathtt{T} \wedge \neg \blacklozenge\, \mathtt{G})$. Intuitively, $\#\mathtt{ch}_{\mathtt{trg}} \geq 2$

can then be defined with the $*$ operator simply as the formula $\#\mathtt{ch}_{\mathtt{trg}} \geq 1 * \#\mathtt{ch}_{\mathtt{trg}} \geq 1$, stating that it is possible to partition the forest into two subforests having both at least one child of t. With the ⧫ operator, this property is instead defined as

$$\#\mathtt{ch}_{\mathtt{trg}} \geq 2 \stackrel{\text{def}}{=} \langle \mathrm{U} \rangle \big(\mathtt{T} \land \neg \blacklozenge \mathtt{G} \land \blacklozenge(\neg\, \mathtt{inDom} \land \#\mathtt{ch}_{\mathtt{trg}} \geq 1)\big).$$

where $\mathtt{inDom} \stackrel{\text{def}}{=} \mathtt{T} \lor \mathtt{G}$ states that the current evaluation node is in the domain of the forest. This definition of $\#\mathtt{ch}_{\mathtt{trg}} \geq 2$ requires to find one child of t (as encoded by the "$\langle \mathrm{U} \rangle(\mathtt{T} \land \neg \blacklozenge \mathtt{G} \land \cdots$" part of the formula) and remove it from the model (as expressed by the "$\blacklozenge(\neg\, \mathtt{inDom} \land \cdots$" part). Only afterwards we check for the existence of a second child of t. This form of "sequential reasoning" (that can be often avoided when using the $*$ operator), is used in almost all the formulae of the next sections: we first find a node satisfying a certain property, we remove it from the structure, and only afterwards we check if the model satisfy a second property. This principle only works well for monotonic properties: with respect to the definition of $\#\mathtt{ch}_{\mathtt{trg}} \geq 2$, the set of children of t monotonically decreases when considering subforests. Thus, finding a child of t in the subforest, implies finding a child of t in the original forest.

## 3 On the complexity and expressive power of ALT

In this section, we show that SAT(ALT) is TOWER-hard by reduction from the satisfiability problem of Propositional Interval Temporal Logic on finite words (Section 3.3). The proof adapts the arguments used in [31] for the version of ALT featuring the separating conjunction $*$. The reduction is somewhat non-intuitive and in [31] it is given without explaining why more direct ways fail. Here, we clarify this issue which is related to the fact that ALT cannot deduce any property of the portion of a pointed forest $(\mathcal{F}, \mathsf{t}, \mathsf{n})$ corresponding to the nodes in $\mathcal{F}(\mathtt{G})$, except for the size of $\mathcal{F}(\mathtt{G})$ and the query $\mathsf{n} \in \mathcal{F}(\mathtt{G})$. This is done in Section 3.2, by relying on a notion of Ehrenfeucht-Fraïssé games for ALT.

### 3.1 Towards the TOWER-hardness of SAT(ALT): how to encode finite words.

As a first step, we define a correspondence between finite words and specific pointed forests. As usual, we define the set of finite words on a finite alphabet $\Sigma$ as the closure under Kleene star $\Sigma^*$. To ease our modelling, we suppose $\Sigma \stackrel{\text{def}}{=} [1, n]$ to be the alphabet of natural numbers between 1 and $n$. Let $\mathfrak{w} = \mathsf{a}_1 \cdots \mathsf{a}_k$ be a $k$-symbols word in $\Sigma^*$ and $\mathsf{M} = \{\mathsf{n}_1, \cdots, \mathsf{n}_k\}$ be a set of $k$ nodes. Let $\mathsf{N}_i$ ($i \in [1, k]$) be a set of $\mathsf{a}_i + 1$ nodes different from $\mathsf{n}_1, \cdots, \mathsf{n}_k$ and so that for each distinct $i, j \in [1, k]$, $\mathsf{N}_i \cap \mathsf{N}_j = \emptyset$. Lastly, let t be a node not in $\mathsf{M} \cup \bigcup_{i \in [1,k]} \mathsf{N}_i$. A pointed forest $(\mathcal{F}, \mathsf{t}, \mathsf{n})$ encodes $\mathfrak{w}$ w.r.t. the sets $\mathsf{M}, \mathsf{N}_1, \cdots, \mathsf{N}_k$ iff (**I**) $\mathcal{F}(\mathsf{n}_k) = \mathsf{t}$, (**II**) for all $i \in [1, k-1]$ $\mathcal{F}(\mathsf{n}_i) = \mathsf{n}_{i+1}$, (**III**) for all $i \in [1, k]$ and $\mathsf{n}' \in \mathsf{N}_i$, $\mathcal{F}(\mathsf{n}') = \mathsf{n}_i$ and (**IV**) every $\mathcal{F}$-descendant of t belongs to a set among $\mathsf{M}, \mathsf{N}_1, \cdots, \mathsf{N}_k$.

We call the path from $\mathsf{n}_1$ to $\mathsf{n}_k$, the *main path* of $\mathcal{F}$. The nodes of this path are the ones in M, and can be characterised as being the only descendants of t with at least one child. Moreover, $\mathsf{n}_1$ is the only node of the main path having the same number of descendants and children. We say that a node $\mathsf{n} \in \mathrm{dom}(\mathcal{F})$ *encodes* the symbol $\mathsf{a} \in \Sigma$ if it is a descendant of t and it has exactly $\mathsf{a} + 1$ children that are not in M. Then, the nodes in M are the only ones encoding symbols, where $\mathsf{n}_i$ encodes $\mathsf{a}_i$ for any $i \in [1, k]$. For instance, Figure 2 shows an encoding of the word 1121.

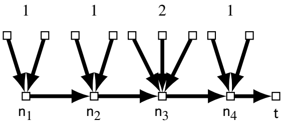

**Fig. 2.** Encoding of 1121.

| Formula | Intended meaning |
|---|---|
| $\texttt{size(G)} \geq \beta$ | $\lvert\mathcal{F}(\texttt{G})_{\mathsf{t}}\rvert \geq \beta$. |
| $\texttt{\#desc} \geq \beta$ | $(\mathcal{F}, \mathsf{t}, \mathsf{n}) \models \mathtt{T}$ and $\mathsf{n}$ has at least $\beta$ descendants. |
| $\texttt{\#child} \geq \beta$ | $(\mathcal{F}, \mathsf{t}, \mathsf{n}) \models \mathtt{T}$ and $\mathsf{n}$ has at least $\beta$ children. |

**Table 1.** Formulae and their meaning on $(\mathcal{F}, \mathsf{t}, \mathsf{n})$.

In order to characterise the class of pointed forests encoding finite words, we adapt the formulae of [31] shown in Table 1 (where their semantics is described). Let $(\mathcal{F}, \mathsf{t}, \mathsf{n})$ be a pointed forest and let $\beta \in \mathbb{N}$. The formula $\texttt{size(G)} \geq \beta$ is inductively defined as:

$$\texttt{size(G)} \geq 0 \stackrel{\text{def}}{=} \mathtt{T}, \qquad \texttt{size(G)} \geq \beta{+}1 \stackrel{\text{def}}{=} \langle \mathrm{U} \rangle \big(\mathtt{G} \wedge \blacklozenge(\neg\, \texttt{inDom} \wedge \texttt{size(G)} \geq \beta)\big).$$

Notice how, in the definition of $\texttt{size(G)} \geq \beta{+}1$, we use the same principle used to encode $\texttt{\#ch}_{\texttt{trg}} \geq 2$ at the end of Section 2: we first find a node in $\mathcal{F}(\texttt{G})$, remove it from the model, and then find other $\beta$ elements of $\mathcal{F}(\texttt{G})$. The formulae $\texttt{\#desc} \geq \beta$ and $\texttt{\#child} \geq \beta$ (again, we refer to Table 1 for their semantics) are instead defined as:

$$\texttt{\#desc} \geq \beta \stackrel{\text{def}}{=} \blacklozenge^*\Big(\underbrace{[\mathrm{U}]\neg\mathtt{G}}_{\mathcal{F}(\texttt{G})\text{ is empty.}} \wedge \mathtt{T} \wedge \underbrace{\blacklozenge(\neg\, \texttt{inDom} \wedge \texttt{size(G)} \geq \beta)}_{\text{Removing } \mathsf{n} \text{ lead to a set of garbage nodes of size at least } \beta.}\Big)$$

$$\texttt{\#child} \geq 0 \stackrel{\text{def}}{=} \mathtt{T}, \qquad \texttt{\#child} \geq \beta{+}1 \stackrel{\text{def}}{=} \texttt{\#desc} \geq \beta{+}1 \wedge \underbrace{\neg \blacklozenge^{\beta}(\mathtt{T} \wedge \neg\, \texttt{\#desc} \geq 1)}_{\text{Whenever } \beta \text{ nodes of } \mathrm{dom}(\mathcal{F}) \text{ are removed, if } \mathsf{n} \text{ still reaches } \mathsf{t} \text{ then it has at least one descendant.}}.$$

Given $\mathtt{s} \in \{\texttt{size(G)}, \texttt{\#ch}_{\texttt{trg}}, \texttt{\#desc}, \texttt{\#child}\}$, we write $\mathtt{s} = \beta$ for $\mathtt{s} \geq \beta \wedge \neg \mathtt{s} \geq \beta{+}1$. For instance, $\texttt{\#child} = \beta$ is the formula that checks whether $\mathsf{n}$ has *exactly* $\beta$ children and it is a descendant of $\mathsf{t}$. We can now conclude the encoding of finite words.

Let $(\mathcal{F}, \mathsf{t}, \mathsf{n})$ be a pointed forest encoding $\mathfrak{w} \in \Sigma^*$ and let $\mathsf{M}$ be the set of nodes in its main path. Let us recall two properties of our encoding: (I) a node $\mathsf{n}'$ encodes a symbol of $\mathfrak{w}$ iff $\mathsf{n}' \in \mathsf{M}$, and (II) the node encoding the first symbol of $\mathfrak{w}$ is the only node in $\mathsf{M}$ with the same number of descendants and children. To reflect (I) we denote with $\texttt{symb}$ the formula $\texttt{\#desc} \geq 1$. For (II), given $\mathsf{S} \subseteq \Sigma$, we introduce the formula $\texttt{1st}_{\mathsf{S}}$ that checks if the current evaluation node corresponds to the first node of the main path and encodes a symbol in $\mathsf{S}$. It is defined as $\bigvee_{\beta \in \mathsf{S}}(\texttt{\#desc} = \beta + 1 \wedge \texttt{\#child} = \beta + 1)$. The following statement formalises the connection between this formula and property (II) stated above.

**Lemma 1.** *Let* $\mathfrak{w} \in \Sigma^+$. *Let* $(\mathcal{F}, \mathsf{t}, \mathsf{n})$ *be a pointed forest encoding* $\mathfrak{w}$. *Let* $\mathsf{n}_1$ *be the first node in the main path of* $\mathcal{F}$. *For every* $\mathsf{S} \subseteq \Sigma$, $(\mathcal{F}, \mathsf{t}, \mathsf{n}) \models \langle \mathrm{U} \rangle\, \texttt{1st}_{\mathsf{S}}$ *iff* $(\mathcal{F}, \mathsf{t}, \mathsf{n}_1) \models \texttt{1st}_{\mathsf{S}}$.

We are finally ready to define the formula $\texttt{word}_{\Sigma}$, characterising the class of forests that encodes words in $\Sigma^*$. It is proved correct by Lemma 2, and it is defined as follows

$$\begin{aligned}\texttt{word}_{\Sigma} \stackrel{\text{def}}{=} &\overbrace{(\langle \mathrm{U} \rangle \mathtt{T} \Rightarrow \langle \mathrm{U} \rangle\, \texttt{symb})}^{\text{The target node has no descendants, or has a descendant that encodes a symbol.}} \wedge \neg\, \texttt{\#ch}_{\texttt{trg}} \geq 2 \wedge \\ &[\mathrm{U}](\texttt{symb} \Rightarrow \texttt{1st}_{\Sigma} \vee \underbrace{(\neg\, \texttt{1st}_{\{n+1\}} \wedge \blacklozenge\, \texttt{1st}_{\Sigma})}_{\text{The current node encodes a symbol in } [1,n] \text{ and exactly one of its children encodes a symbol.}}).\end{aligned}$$

**Lemma 2.** *A pointed forest* $(\mathcal{F}, \mathsf{t}, \mathsf{n})$ *is an encoding of a word in* $\Sigma^*$ *iff* $(\mathcal{F}, \mathsf{t}, \mathsf{n}) \models \texttt{word}_{\Sigma}$.

**game played on** $((\mathcal{F}_1, \mathsf{t}_1, \mathsf{n}_1), (\mathcal{F}_2, \mathsf{t}_2, \mathsf{n}_2), (\mathsf{m}, \mathsf{s}, \mathsf{k}))$
**if** there is $p \in \{\mathtt{G}, \mathtt{T}\}$ s.t. not $((\mathcal{F}_1, \mathsf{t}_1, \mathsf{n}_1) \models p$ iff $(\mathcal{F}_2, \mathsf{t}_2, \mathsf{n}_2) \models p)$ **then** the spoiler wins, **otherwise** the spoiler chooses $i \in \{1, 2\}$ and plays on $(\mathcal{F}_i, \mathsf{t}_i, \mathsf{n}_i)$. The duplicator replies on $(\mathcal{F}_j, \mathsf{t}_j, \mathsf{n}_j)$ where $j \in \{1, 2\} \setminus \{i\}$. The spoiler **must** choose one of the following moves (else the duplicator wins).

$\langle \mathrm{U} \rangle$ **move**: if $\mathsf{m} \geq 1$ then the spoiler **can** choose to play a $\langle \mathrm{U} \rangle$ move. It selects a node $\mathsf{n}'_i \in \mathcal{N}$.

- Then, the duplicator **must** reply with some node $\mathsf{n}'_j \in \mathcal{N}$ (otherwise the spoiler wins).
- The game continues on $((\mathcal{F}_1, \mathsf{t}_1, \mathsf{n}'_1), (\mathcal{F}_2, \mathsf{t}_2, \mathsf{n}'_2), (\mathsf{m}-1, \mathsf{s}, \mathsf{k}))$.

$\blacklozenge$ **move**: if $\mathsf{s} \geq 1$ and $\mathrm{dom}(\mathcal{F}_i) \neq \emptyset$ then the spoiler **can** choose to play a $\blacklozenge$ move. It selects a finite forest $\mathcal{F}'_i$ such that $\mathcal{F}'_i \sqsubseteq \mathcal{F}_i$ and $|\mathrm{dom}(\mathcal{F}'_i)| = |\mathrm{dom}(\mathcal{F}_i)| - 1$.

- The duplicator **must** reply with some $\mathcal{F}'_j \sqsubseteq \mathcal{F}_j$ s.t. $|\mathrm{dom}(\mathcal{F}'_j)| = |\mathrm{dom}(\mathcal{F}_j)| - 1$.
- The game continues on $((\mathcal{F}'_1, \mathsf{t}_1, \mathsf{n}_1), (\mathcal{F}'_2, \mathsf{t}_2, \mathsf{n}_2), (\mathsf{m}, \mathsf{s}-1, \mathsf{k}))$.

$\blacklozenge^*$ **move**: if $\mathsf{k} \geq 1$ then the spoiler **can** choose to play a $\blacklozenge^*$ move. It selects a forest $\mathcal{F}'_i \sqsubseteq \mathcal{F}_i$.

- The duplicator **must** reply with some $\mathcal{F}'_j$ s.t. $\mathcal{F}'_j \sqsubseteq \mathcal{F}_j$.
- The game continues on $((\mathcal{F}'_1, \mathsf{t}_1, \mathsf{n}_1), (\mathcal{F}'_2, \mathsf{t}_2, \mathsf{n}_2), (\mathsf{m}, \mathsf{s}, \mathsf{k}-1))$.

**Fig. 3.** Ehrenfeucht-Fraïssé games for ALT

### 3.2 Inexpressibility results via the Ehrenfeucht-Fraïssé games for ALT

Now that we are more familiar with the logic, before completing the TOWER-hardness proof of SAT(ALT) we show some properties that ALT cannot express. Notably, these properties explain why the TOWER-hardness proof of the next section cannot be easily simplified. Moreover, inexpressibility results effectively reduce the set of forests that must be considered in order to solve SAT(ALT). This in turn makes reductions from SAT(ALT) to other logics more immediate, as we show throughout Section 4.

A standard way of proving inexpressibility results for logics interpreted on finite models is by adaptation of the Ehrenfeucht-Fraïssé games [29], as done for other relation-changing logics such as context logic for trees [10] and ambient logic [16].

We define the *rank* of a formula $\varphi$ as the triple $(\mathsf{m}, \mathsf{s}, \mathsf{k}) \in \mathbb{N}^3$ where the *modal rank* $\mathsf{m}$ is the greatest nesting depth of the modal operator $\langle \mathrm{U} \rangle$ in $\varphi$, whereas the *sabotage rank* $\mathsf{s}$ (resp. *repeated sabotage rank* $\mathsf{k}$) is the greatest nesting depth of the $\blacklozenge$ (resp. $\blacklozenge^*$) operator in $\varphi$. We denote with $\mathsf{ALT}(\mathsf{rk})$ the set of formulae with rank $\mathsf{rk} \in \mathbb{N}^3$.

The Ehrenfeucht-Fraïssé games (EF-games) for ALT are formally defined in Figure 3. A game is played by two players: the *spoiler* and the *duplicator*. A game state $((\mathcal{F}_1, \mathsf{t}_1, \mathsf{n}_1), (\mathcal{F}_2, \mathsf{t}_2, \mathsf{n}_2), \mathsf{rk})$ is a triple made of a rank $\mathsf{rk}$ and two pointed forests $(\mathcal{F}_1, \mathsf{t}_1, \mathsf{n}_1)$ and $(\mathcal{F}_2, \mathsf{t}_2, \mathsf{n}_2)$. The goal of the spoiler is to show that the two structures are different. The goal of the duplicator is to counter the spoiler and show that the two structures are similar. Let us make clear what we mean by two models being different: both players can only play following the rules of the logical formalism (in our case, ALT). Then, two models are different if and only if there is a formula $\varphi \in \mathsf{ALT}(\mathsf{rk})$ that it is satisfied by only one of the two models. This correspondence between the game and the logic is expressed by an adequacy result, formalised below in Lemma 3.

A player has a *winning strategy* if it can play in a way that guarantees it the victory, regardless what the other player does. We write $(\mathcal{F}_1, \mathsf{t}_1, \mathsf{n}_1) \approx_{\mathsf{rk}} (\mathcal{F}_2, \mathsf{t}_2, \mathsf{n}_2)$ whenever the duplicator has a winning strategy for the game $((\mathcal{F}_1, \mathsf{t}_1, \mathsf{n}_1), (\mathcal{F}_2, \mathsf{t}_2, \mathsf{n}_2), \mathsf{rk})$. By Martin's Theorem [32] our games are determined: if the duplicator does not have a winning strategy then spoiler has one, and vice-versa. Hence, $(\mathcal{F}_1, \mathsf{t}_1, \mathsf{n}_1) \not\approx_{\mathsf{rk}} (\mathcal{F}_2, \mathsf{t}_2, \mathsf{n}_2)$ refers to the fact that the spoiler has a winning strategy.

**Lemma 3.** $(\mathcal{F}_1, \mathsf{t}_1, \mathsf{n}_1) \not\approx_{\mathsf{rk}} (\mathcal{F}_2, \mathsf{t}_2, \mathsf{n}_2)$ *iff* $\exists \varphi \in \mathsf{ALT}(\mathsf{rk})$, $(\mathcal{F}_1, \mathsf{t}_1, \mathsf{n}_1) \models \varphi$ *and* $(\mathcal{F}_2, \mathsf{t}_2, \mathsf{n}_2) \not\models \varphi$.

The left-to-right direction is proved by induction on the rank and by cases on the first move that the spoiler makes in his winning strategy. The other direction is proved by structural induction on $\varphi$. We start to use the EF-games to derive three easy results.

**Lemma 4.** *Let $\varphi$ be a formula.*

1. *$\varphi$ is satisfiable iff it is satisfiable by a pointed forest* $(\mathcal{F}, \mathsf{t}, \mathsf{n})$ *where* $\mathsf{t} \notin \mathrm{dom}(\mathcal{F})$.
2. *Given a forest $\mathcal{F}$ and nodes* $\mathsf{t} \in \mathcal{N}$ *and* $\mathsf{n}, \mathsf{n}' \notin \mathrm{dom}(\mathcal{F})$, $(\mathcal{F}, \mathsf{t}, \mathsf{n}) \models \varphi$ *iff* $(\mathcal{F}, \mathsf{t}, \mathsf{n}') \models \varphi$.
3. *If duplicator has a winning strategy for a game* $((\mathcal{F}_1, \mathsf{t}_1, \mathsf{n}_1), (\mathcal{F}_2, \mathsf{t}_2, \mathsf{n}_2), \mathsf{rk})$ *then it has a winning strategy where it always replies to* $\langle \mathrm{U} \rangle$ *moves by selecting nodes in* $\mathrm{dom}(\mathcal{F}_i) \cup \mathrm{ran}(\mathcal{F}_i)$, *for some* $i \in \{1, 2\}$.

*Proof (sketch).* We sketch the proof of (1) to show how EF-games are used. Let us consider a pointed forest $(\mathcal{F}, \mathsf{t}, \mathsf{n})$ such that $(\mathcal{F}, \mathsf{t}, \mathsf{n}) \models \varphi$. We take a node $\mathsf{t}' \notin \mathrm{dom}(\mathcal{F}) \cup \mathrm{ran}(\mathcal{F})$ and define the forest $\mathcal{F}'(\mathsf{n}') \stackrel{\text{def}}{=}$ **if** $\mathcal{F}(\mathsf{n}') = \mathsf{t}$ **then** $\mathsf{t}'$ **else** $\mathcal{F}(\mathsf{n}')$. Notice that $\mathsf{t}' \notin \mathrm{dom}(\mathcal{F}')$. We then prove $\forall \mathsf{rk} \in \mathbb{N}^3$ $(\mathcal{F}, \mathsf{t}, \mathsf{n}) \approx_{\mathsf{rk}} (\mathcal{F}', \mathsf{t}', \mathsf{n})$ by induction on $\mathsf{rk}$, leading to (1) directly by Lemma 3. The proof of (3) essentially follows from (2). □

Interestingly enough, the third statement of Lemma 4 fundamentally implies that enforcing $\mathcal{N}$ to be finite, instead of infinite as we do throughout this work, does not change the expressive power nor the complexity of ALT.

Let $(\mathcal{F}, \mathsf{t}, \mathsf{n})$ be a pointed forest. We now show that ALT has a very limited expressive power with respect to the garbage nodes. In particular, it can only check for the membership of $\mathsf{n}$ in $\mathcal{F}(\mathtt{G})$ (with the formula $\mathtt{G}$) and for the size of $\mathcal{F}(\mathtt{G})$ (with the formula $\mathtt{size}(\mathtt{G}) \geq \beta$). We formalise this inexpressibility result as follows.

**Lemma 5.** *Let* $\mathsf{rk} = (\mathsf{m}, \mathsf{s}, \mathsf{k})$. *Let $\mathcal{F}, \mathcal{F}_1$ and $\mathcal{F}_2$ be three forests and let* $\mathsf{n}, \mathsf{t} \in \mathcal{N}$, *such that for every $i \in \{1, 2\}$, $\mathcal{F} \sqsubseteq \mathcal{F}_i$ and* $\mathcal{F}_i(\mathtt{G})_{\mathsf{t}} = \mathrm{dom}(\mathcal{F}_i) \setminus \mathrm{dom}(\mathcal{F})$. *If we have*

$$\mathsf{n} \in \mathcal{F}_1(\mathtt{G})_{\mathsf{t}} \textit{ iff } \mathsf{n} \in \mathcal{F}_2(\mathtt{G})_{\mathsf{t}} \quad \textit{and} \quad \min(|\mathcal{F}_1(\mathtt{G})_{\mathsf{t}}|, \mathsf{m} + \mathsf{s} + \mathsf{k}) = \min(|\mathcal{F}_2(\mathtt{G})_{\mathsf{t}}|, \mathsf{m} + \mathsf{s} + \mathsf{k})$$

*then* $(\mathcal{F}_1, \mathsf{t}, \mathsf{n}) \approx_{\mathsf{rk}} (\mathcal{F}_2, \mathsf{t}, \mathsf{n})$.

Let us informally explain Lemma 5, whose proof is by induction on $\mathsf{rk}$ and by cases on the moves of the spoiler. Let $(\mathcal{F}_1, \mathsf{t}, \mathsf{n})$ be a pointed forest and suppose (ad absurdum) that it satisfies a formula $\varphi$ of rank $\mathsf{rk}$ that express a property of the garbage nodes that is different form the ones cited above. For example, let us assume that $\varphi$ characterise the set of pointed forests having a garbage node with at least two children. Consider the subforest $\mathcal{F} \sqsubseteq \mathcal{F}_1$ whose domain corresponds to the set of $\mathcal{F}_1$-descendants of $\mathsf{t}$. In particular, $\mathcal{F}_1(\mathtt{G})_{\mathsf{t}} = \mathrm{dom}(\mathcal{F}_1) \setminus \mathrm{dom}(\mathcal{F})$. We extend $\mathcal{F}$ to a forest $\mathcal{F}_2$ by (re)defining it on the nodes in $\mathcal{F}_1(\mathtt{G})_{\mathsf{t}}$ so that $\mathcal{F}_2(\mathtt{G})_{\mathsf{t}} = \mathcal{F}_1(\mathtt{G})_{\mathsf{t}}$ and none of these nodes has more than one $\mathcal{F}_2$-child (this construction can always be done). This last equality implies that $\mathsf{n} \in \mathcal{F}_1(\mathtt{G})_{\mathsf{t}} \Leftrightarrow \mathsf{n} \in \mathcal{F}_2(\mathtt{G})_{\mathsf{t}}$ and $\min(|\mathcal{F}_1(\mathtt{G})_{\mathsf{t}}|, \mathsf{m} + \mathsf{s} + \mathsf{k}) = \min(|\mathcal{F}_2(\mathtt{G})_{\mathsf{t}}|, \mathsf{m} + \mathsf{s} + \mathsf{k})$. By Lemma 5 $(\mathcal{F}_1, \mathsf{t}, \mathsf{n}) \approx_{\mathsf{rk}} (\mathcal{F}_2, \mathsf{t}, \mathsf{n})$, which implies $(\mathcal{F}_2, \mathsf{t}, \mathsf{n}) \models \varphi$ by Lemma 3. However, $(\mathcal{F}_2, \mathsf{t}, \mathsf{n})$ is defined so that every node in $\mathcal{F}_2(\mathtt{G})_{\mathsf{t}}$ has at most one child. Thus, $\varphi$ cannot characterise the set of models having a garbage node with at least two children.

As shown in the next section, the inexpressibility result in Lemma 5 plays a central role in the development of the reduction that leads to the TOWER-hardness of SAT(ALT).

### 3.3 PITL on marked words and the TOWER-hardness of SAT(ALT)

We are now ready to show the non-elementarity of SAT(ALT). The proof is by reduction from the satisfiability problem of Propositional Interval Temporal Logic (PITL) under locality principle [34,25], which in turn is shown TOWER-hard by reduction from the non-emptiness problem of star-free regular languages (see [38] for the TOWER characterisation of this problem). PITL is a well-known logic that was introduced by Moszkowski in [34] for the verification of hardware components. It is interpreted on non-empty finite words over a finite alphabet of unary symbols $\Sigma$. Its formulae are from the grammar:

$$\varphi := \varphi \wedge \varphi \mid \neg\varphi \mid \mathsf{a} \mid 1 \mid \varphi \,|\, \varphi$$

where $\mathsf{a} \in \Sigma$. Under the *locality principle* interpretation, a word $\mathfrak{w} = \mathsf{a}_1 \cdots \mathsf{a}_k \in \Sigma^+$ satisfies $\mathsf{a}$ whenever $\mathsf{a}_1 = \mathsf{a}$. Moreover, $\mathfrak{w}$ satisfies $1$ if it is a word of length one (i.e. $\mathfrak{w} \in \Sigma$). The main feature of this logic is its *chop* operator "$|$". Intuitively, $\varphi|\psi$ is satisfied by words that can be "chopped" into a prefix and a suffix sharing one symbol, so that the prefix satisfies $\varphi$ and the suffix satisfies $\psi$. Formally,

$$\mathsf{a}_1 \cdots \mathsf{a}_k \models \varphi|\psi \quad \overset{\text{def}}{\Leftrightarrow} \quad \text{there is } i \in [1,k] \text{ such that } \mathsf{a}_1 \cdots \mathsf{a}_i \models \varphi \text{ and } \mathsf{a}_i \cdots \mathsf{a}_k \models \psi.$$

Translating $|$ in ALT is not easy. Indeed, given the encoding of words proposed in Section 3.1, chopping $\mathfrak{w}$ in two pieces means splitting in some way the main path $\mathsf{n}_1, \cdots, \mathsf{n}_k$ of a forest $(\mathcal{F}, \mathsf{t}, \mathsf{n})$ encoding $\mathfrak{w}$ to then check that the word encoded by $\mathsf{n}_1, \cdots, \mathsf{n}_i$ satisfies $\varphi$ and the one encoded by $\mathsf{n}_i, \cdots, \mathsf{n}_k$ satisfies $\psi$. However, by doing this the elements $\mathsf{n}_1, \cdots, \mathsf{n}_i$ become garbage nodes. Thus, as a consequence of Lemma 5, ALT cannot check in any way what is the word encoded by these nodes. Easy translations from PITL to ALT seem therefore impossible and, as done in [31], we are required to go through an alternative interpretation of PITL based on *marking symbols* instead of chopping words.

A *marking* of an alphabet $\Sigma$ is a bijection $\overline{(.)} : \Sigma \to \overline{\Sigma}$, relating a symbol $\mathsf{a} \in \Sigma$ to its *marked variant* $\bar{\mathsf{a}} \in \overline{\Sigma}$. We denote with $\mathbb{\Sigma}$ the extended alphabet $\Sigma \uplus \overline{\Sigma}$. A word is *marked* if it has some symbols from $\overline{\Sigma}$. We introduce the satisfaction relation $\models_\bullet$ on a marked word $\mathfrak{w} \in \mathbb{\Sigma}^+$. It is defined as usual for Boolean connectives. Moreover,

$$\mathfrak{w} \models_\bullet \mathsf{a} \overset{\text{def}}{\Leftrightarrow} \mathfrak{w} \text{ is headed by } \mathsf{a} \text{ or } \bar{\mathsf{a}}; \qquad \mathfrak{w} \models_\bullet 1 \overset{\text{def}}{\Leftrightarrow} \mathfrak{w} \text{ is headed by a marked symbol.}$$

The definition of $\varphi|\psi$ is more involved. Let $\mathfrak{w}' \in \Sigma^*$, $\bar{\mathsf{a}} \in \overline{\Sigma}$ and $\mathfrak{w}'' \in \mathbb{\Sigma}^*$ be such that $\mathfrak{w} = \mathfrak{w}'\bar{\mathsf{a}}\mathfrak{w}''$, so that $\bar{\mathsf{a}}$ is the first marked symbol occurring in $\mathfrak{w}$ (this decomposition is uniquely defined). Then, $\mathfrak{w}'\bar{\mathsf{a}}\mathfrak{w}'' \models_\bullet \varphi|\psi$ holds if and only if there is there is $\mathsf{b} \in \Sigma$ s.t.

(a) $\mathfrak{w}'$ is the empty word, $\mathsf{b} = \mathsf{a}$ and $\bar{\mathsf{a}}\mathfrak{w}'' \models_\bullet \varphi \wedge \psi$, or
(b) there is $\mathfrak{w}_2 \in \Sigma^*$ s.t. $\mathfrak{w}' = \mathsf{b}\mathfrak{w}_2$, $\bar{\mathsf{b}}\mathfrak{w}_2\bar{\mathsf{a}}\mathfrak{w}'' \models \varphi$ and $\mathsf{b}\mathfrak{w}_2\bar{\mathsf{a}}\mathfrak{w}'' \models \psi$, or
(c) $\mathfrak{w}'$ is not the empty word, $\mathsf{b} = \mathsf{a}$, $\mathfrak{w}'\bar{\mathsf{a}}\mathfrak{w}'' \models \varphi$ and $\bar{\mathsf{a}}\mathfrak{w}'' \models \psi$, or
(d) $\exists \mathfrak{w}_1 \in \Sigma^+, \exists \mathfrak{w}_2 \in \Sigma^*$ s.t. $\mathfrak{w}' = \mathfrak{w}_1\mathsf{b}\mathfrak{w}_2$, $\mathfrak{w}_1\bar{\mathsf{b}}\mathfrak{w}_2\bar{\mathsf{a}}\mathfrak{w}'' \models \varphi$ and $\mathsf{b}\mathfrak{w}_2\bar{\mathsf{a}}\mathfrak{w}'' \models \psi$.

On this semantics, the satisfaction of a formula only depends on the prefix $\mathsf{a}_1 \cdots \mathsf{a}_{i-1}\bar{\mathsf{a}}_i$ of $\mathfrak{w}$ that ends with the first marked symbol. To check whether $\mathfrak{w} \models_\bullet \varphi|\psi$ we search for a position $j \in [1,i]$ inside this prefix so that $\varphi$ is satisfied by the word obtained from $\mathfrak{w}$ by marking the $j$-th symbol, whereas $\psi$ is satisfied by the suffix of $\mathfrak{w}$ starting in $j$. In the definition above, this idea is split into four cases (a)–(d), depending on truthiness of $j = 1$ and $j = i$. This is done as it better reflects the encoding of PITL in ALT. The semantics on marked words is related to the standard semantics of PITL as follows.

**Proposition 1 (from [31]).** *Let* $\mathfrak{w} \in \Sigma^*$, $\mathsf{a} \in \Sigma$ *and* $\mathfrak{w}' \in \mathbb{\Sigma}^*$. *Let* $\varphi$ *be a formula in PITL.* $\mathfrak{w}\mathsf{a}$ *satisfies* $\varphi$ *under the standard interpretation of PITL if and only if* $\mathfrak{w}\bar{\mathsf{a}}\mathfrak{w}' \models_{\bullet} \varphi$.

The alternative interpretation of PITL allows us to reduce SAT(PITL) to SAT(ALT) in a neat way. Let $\Sigma = [1, n]$, $\mathbb{\Sigma} = \Sigma \cup \overline{\Sigma}$ and let $\mathfrak{f} : \mathbb{\Sigma} \to [1, 2n]$ be the bijection $\mathfrak{f}(\mathsf{a}) \stackrel{\text{def}}{=} 2\mathsf{a}$ for $\mathsf{a} \in \Sigma$ and $\mathfrak{f}(\bar{\mathsf{a}}) \stackrel{\text{def}}{=} 2\mathsf{a} - 1$ for $\bar{\mathsf{a}} \in \overline{\Sigma}$. $\mathfrak{f}(\mathsf{a}_1 \cdots \mathsf{a}_k)$ denotes the word $\mathfrak{f}(\mathsf{a}_1) \cdots \mathfrak{f}(\mathsf{a}_k)$. $\mathfrak{f}$ maps $\mathbb{\Sigma}$ into the alphabet $[1, 2n]$, whose words can be encoded into trees (as in Section 3.1). In these trees each symbol $\mathsf{a} \in \Sigma$ (resp. $\bar{\mathsf{a}} \in \overline{\Sigma}$) corresponds to a node in the main path having $2\mathsf{a} + 1$ (resp. $2\mathsf{a}$) children not in this path. Therefore, given a node $\mathsf{n}$ encoding a symbol in $\Sigma$, removing exactly one children of $\mathsf{n}$ that is not in the main path is equivalent to marking the symbol $\mathsf{n}$ encodes. Based on this description, we can check if the current evaluation node encodes a marked symbol from $\overline{\Sigma}$ with the following formula:

$$\mathtt{mark}_{\Sigma} \stackrel{\text{def}}{=} \bigvee_{\mathsf{a} \in \Sigma} \big((\#\mathtt{child} = 2\mathsf{a} \land \mathtt{1st}_{[1,2n]}) \lor (\#\mathtt{child} = 2\mathsf{a} + 1 \land \neg\, \mathtt{1st}_{[1,2n]})\big)$$

As already stated, $\mathfrak{w} \models_{\bullet} \varphi$ examines the prefix of $\mathfrak{w}$ that ends with the first marked symbol. In pointed forests $(\mathcal{F}, \mathsf{t}, \mathsf{n})$ encoding $\mathfrak{w}$, this prefix corresponds to the subtree whose root encodes a marked symbol and is a $\mathcal{F}$-descendant of every other node encoding marked symbols. Therefore, to characterise this tree we need to track the number of nodes encoding marked symbols. We first define a formula $\mathtt{marks}_{\Sigma} \geq \beta$ stating that the forest has at least $\beta \in \mathbb{N}$ nodes encoding marked symbols. It is defined as $\top$ for $\beta = 0$, and otherwise ($\beta \geq 1$) as $\langle \mathsf{U} \rangle \big(\mathtt{mark}_{\Sigma} \land \blacklozenge(\neg\, \mathtt{inDom} \land \mathtt{marks}_{\Sigma} \geq \beta - 1)\big)$. Again, this formula uses the same principle introduced in Section 2 for $\#\mathtt{ch}_{\mathtt{trg}} \geq 2$: we search for a node encoding a marked symbol, remove it from the structure and then search for $\beta - 1$ other such nodes. Similarly, we introduce $\#\mathtt{markAnc}_{\Sigma} \geq \beta \stackrel{\text{def}}{=} \mathtt{symb} \land \blacklozenge(\neg\, \mathtt{inDom} \land \mathtt{marks}_{\Sigma} \geq \beta)$, the formula stating that the current evaluation node encodes a symbol and has at least $\beta$ ancestors that encode marked symbols.

At last, for a formula $\varphi$ in PITL having symbols from $\Sigma = [1, n]$, we introduce its translation $\nabla_{\beta}(\varphi)$ in ALT, where $\beta \geq 1$ tracks the number of nodes encoding marked symbols. It is homomorphic for Boolean connectives: $\nabla_{\beta}(\neg\varphi) \stackrel{\text{def}}{=} \neg\nabla_{\beta}(\varphi)$ and $\nabla_{\beta}(\varphi \land \psi) \stackrel{\text{def}}{=} \nabla_{\beta}(\varphi) \land \nabla_{\beta}(\psi)$. For $\mathsf{a} \in \Sigma$ and $1$, it faithfully represent the $\models_{\bullet}$ relation: $\nabla_{\beta}(\mathsf{a}) \stackrel{\text{def}}{=} \langle \mathsf{U} \rangle\, \mathtt{1st}_{[2\mathsf{a}-1, 2\mathsf{a}]}$ and $\nabla_{\beta}(\mathtt{1}) \stackrel{\text{def}}{=} \langle \mathsf{U} \rangle (\mathtt{1st}_{[1,2n]} \land \mathtt{mark}_{\Sigma})$. Lastly, the formula $\nabla_{\beta}(\varphi \,|\, \psi)$ is defined as

$$\begin{aligned}
&\langle \mathsf{U} \rangle \big(\, \mathtt{symb} \land \big((\mathtt{1st}_{[1,2n]} \land \mathtt{mark}_{\Sigma} \land \nabla_{\beta}(\varphi) \land \nabla_{\beta}(\psi)) \lor \\
&\ (\mathtt{1st}_{[1,2n]} \land \neg\, \mathtt{mark}_{\Sigma} \land \blacklozenge(\mathtt{mark}_{\Sigma} \land \nabla_{\beta+1}(\varphi)) \land \nabla_{\beta}(\psi)) \lor \\
&\ (\neg\, \mathtt{1st}_{[1,2n]} \land \mathtt{mark}_{\Sigma} \land \#\mathtt{markAnc}_{\Sigma} \geq \beta - 1 \land \nabla_{\beta}(\varphi) \land \blacklozenge(\mathtt{1st}_{[1,2n]} \land \nabla_{\beta}(\psi))) \lor \\
&\ (\neg\, \mathtt{1st}_{[1,2n]} \land \neg\, \mathtt{mark}_{\Sigma} \land \#\mathtt{markAnc}_{\Sigma} \geq \beta \land \blacklozenge(\mathtt{mark}_{\Sigma} \land \nabla_{\beta+1}(\varphi)) \land \blacklozenge(\mathtt{1st}_{[1,2n]} \land \nabla_{\beta}(\psi)))\big)\big).
\end{aligned}$$

Notice how $\nabla_{\beta}(\varphi \,|\, \psi)$ follows closely the $\models_{\bullet}$ relation: it is split into four disjuncts, one for each case in the definition of $\varphi \,|\, \psi$. For example, the second disjunct of $\nabla_{\beta}(\varphi \,|\, \psi)$ encodes the case (b) in the definition of $\mathfrak{w}'\bar{\mathsf{a}}\mathfrak{w}'' \models_{\bullet} \varphi \,|\, \psi$, as schematised below:

| PITL | $\exists \mathsf{b} \in \Sigma \ldots$ | $\exists \mathfrak{w}_2 \in \Sigma^*$ s.t. $\mathfrak{w}' = \mathsf{b}\mathfrak{w}_2$ | and $\bar{\mathsf{b}}\mathfrak{w}_2\bar{\mathsf{a}}\mathfrak{w}'' \models \varphi$ | and $\mathsf{b}\mathfrak{w}_2\bar{\mathsf{a}}\mathfrak{w}'' \models \psi$ |
|---|---|---|---|---|
| ALT | $\langle \mathsf{U} \rangle(\mathtt{symb} \ldots$ | $\mathtt{1st}_{[1,2n]} \land \neg\, \mathtt{mark}_{\Sigma}$ | $\land \blacklozenge(\mathtt{mark}_{\Sigma} \land \nabla_{\beta+1}(\varphi))$ | $\land \nabla_{\beta}(\psi)$ |

The translation is proved correct (by induction on the structure of $\varphi$) in the next lemma.

**Lemma 6.** *Let* $\Sigma = [1, n]$ *and* $\mathbb{\Sigma} = \Sigma \cup \overline{\Sigma}$. *Let* $\mathfrak{w} \in \mathbb{\Sigma}^+$ *with* $\beta \geq 1$ *marked symbols. Let* $(\mathcal{F}, \mathsf{t}, \mathsf{n})$ *be an encoding of* $\mathfrak{f}(\mathfrak{w})$. *For every* $\varphi$ *in PITL,* $\mathfrak{w} \models_{\bullet} \varphi$ *iff* $(\mathcal{F}, \mathsf{t}, \mathsf{n}) \models \nabla_{\beta}(\varphi)$.

Then, the reduction from SAT(PITL) on standard semantics follows as we are able to characterise the set of pointed forests encoding words in $\Sigma^*\overline{\Sigma}$ (first three conjuncts in the formula of Lemma 7). To conclude, we simply apply Lemma 6 and Proposition 1.

**Lemma 7.** *Every $\varphi$ in PITL written with symbols from $\Sigma = [1, n]$ is satisfiable under the standard interpretation of PITL if and only if the following formula in* ALT *is satisfiable*

$$\underbrace{\mathtt{word}_{[1,2n]} \land \langle \mathrm{U}\rangle \top \land [\mathrm{U}](\mathtt{mark}_{\Sigma} \Leftrightarrow \top \land \neg \blacklozenge(\mathrm{G}))}_{\text{The forest encodes a non-empty word. The only node encoding a marked symbol is the child of the target node.}} \land \nabla_1(\varphi).$$

Because of the case distinction in the formula $\nabla_\beta(\varphi \,|\, \psi)$, the formula obtained via $\nabla_\beta$ is exponential (hence elementary) in the number of symbols used to write $\varphi$. Therefore, from the TOWER-hardness of SAT(PITL) we conclude that SAT(ALT) is TOWER-hard.

## 4 Revisiting TOWER-hard logics with ALT

We now display the usefulness of ALT as a tool for proving the TOWER-hardness of logics interpreted on tree-like structures. In particular, we provide semantically faithful reductions from SAT(ALT) to the satisfiability problem of four logics that were independently found to be TOWER-complete: first-order separation logic [9], quantified CTL on trees [28], modal logic of heaps [17] and modal separation logic [18]. Our reduction only use strict fragments of these formalisms, allowing us to draw some new results on these logics. Most notably, this section shows that all these logics are TOWER-hard because they fundamentally provide the reachability and submodel reasoning given by ALT.

### 4.1 From ALT to First-Order Separation Logic

Separation logic (SL) [37] is an assertion logic used in state-of-the-art tools [6,11] for Hoare-style verification of heap-manipulating programs. As already stated, a preliminary definition of ALT was defined in [31] to reason on the complexity of separation logic. Hence, here we briefly revisit the relation between ALT and SL.

Let VAR and LOC be two countably infinite sets of program variables and locations, respectively. Separation logic is interpreted on *memory states*: pairs $(s, h)$ consisting of a function (the *store*) $s : \mathsf{VAR} \to \mathsf{LOC}$ and a partial function with finite domain (the *heap*) $h : \mathsf{LOC} \to_{\mathtt{fin}} \mathsf{LOC}$. Since $\mathcal{N}$ and LOC are both countably infinite sets, w.l.o.g. we assume $\mathsf{LOC} = \mathcal{N}$. We extend the notation of domain, image and function composition to stores and heaps. Two heaps $h_1$ and $h_2$ are said to be disjoint, written $h_1 \perp h_2$, whenever $\mathrm{dom}(h_1) \cap \mathrm{dom}(h_2) = \emptyset$, and when this holds the union $h_1 + h_2$ of $h_1$ and $h_2$ is defined as the standard sum of functions $(h_1 + h_2)(\ell) \stackrel{\text{def}}{=}$ **if** $\ell \in \mathrm{dom}(h_1)$ **then** $h_1(\ell)$ **else** $h_2(\ell)$. Let $\mathtt{u} \in \mathsf{VAR}$ be a *fixed variable* that is reserved for quantification (quantification over other variables is not possible). We consider the separation logic $1\mathsf{SL}(*, \mathtt{alloc}, \hookrightarrow^+)$, whose formulae are built from the following grammar (as in [31]):

$$\varphi := \top \mid \varphi \land \varphi \mid \neg\varphi \mid \mathtt{emp} \mid \mathtt{x} = \mathtt{y} \mid \mathtt{x} \hookrightarrow \mathtt{y} \mid \mathtt{alloc}(\mathtt{x}) \mid \mathtt{x} \hookrightarrow^+ \mathtt{y} \mid \varphi * \varphi \mid \exists \mathtt{u}\, \varphi$$

where $\mathtt{x}, \mathtt{y} \in \mathsf{VAR}$. As shown below, the *reachability predicate* $\hookrightarrow^+$ can be seen as the transitive closure of the standard *points-to* predicate $\hookrightarrow$ of separation logic. For a memory state $(s, h)$, the satisfaction relation $\models$ is defined as follows:

$$\begin{array}{llll} (s,h) \models \mathtt{emp} & \overset{\text{def}}{\Leftrightarrow} \mathrm{dom}(h) = \emptyset. & (s,h) \models \mathtt{x} \hookrightarrow \mathtt{y} & \overset{\text{def}}{\Leftrightarrow} h(s(\mathtt{x})) = s(\mathtt{y}). \\ (s,h) \models \mathtt{x} = \mathtt{y} & \overset{\text{def}}{\Leftrightarrow} s(\mathtt{x}) = s(\mathtt{y}). & (s,h) \models \mathtt{alloc}(\mathtt{x}) & \overset{\text{def}}{\Leftrightarrow} s(\mathtt{x}) \in \mathrm{dom}(h). \\ (s,h) \models \mathtt{x} \hookrightarrow^{+} \mathtt{y} & \overset{\text{def}}{\Leftrightarrow} \text{there is } \exists \delta \geq 1 \text{ such that } h^{\delta}(s(\mathtt{x})) = s(\mathtt{y}). && \\ (s,h) \models \varphi * \psi & \overset{\text{def}}{\Leftrightarrow} \exists h_1, h_2 \text{ s.t. } h_1 \perp h_2,\ h_1 + h_2 = h,\ (s,h_1) \models \varphi \text{ and } (s,h_2) \models \psi. && \\ (s,h) \models \exists \mathtt{u}\ \varphi & \overset{\text{def}}{\Leftrightarrow} \text{there is a location } \ell' \in \mathsf{LOC} \text{ such that } (s[\mathtt{u} \leftarrow \ell'], h) \models \varphi, && \end{array}$$

where $s[\mathtt{u} \leftarrow \ell']$ is the store updated from $s$ by only changing the evaluation of u from $s(\mathtt{u})$ to $\ell'$, i.e. for every $\mathtt{x} \in \mathsf{VAR}$, $s[\mathtt{u} \leftarrow \ell'](\mathtt{x}) \overset{\text{def}}{=}$ **if** $\mathtt{x} = \mathtt{u}$ (syntactically) **then** $\ell'$ **else** $s(\mathtt{x})$. The main ingredient of separation logic is the *separating conjunction* $\varphi * \psi$, that is satisfied whether $h$ can be partitioned into $h_1$ and $h_2$ so that $(s, h_1) \models \varphi$ whereas $(s, h_2) \models \psi$. The $*$ operator captures the $\blacklozenge$ and $\blacklozenge^*$ operators as follows. Consider the formula $\mathtt{size} = 1 \overset{\text{def}}{=} \neg\mathtt{emp} \wedge \neg(\neg\mathtt{emp} * \neg\mathtt{emp})$, which is satisfied whenever $|\mathrm{dom}(h)| = 1$. We define $\blacklozenge_{\text{SL}} \varphi \overset{\text{def}}{=} (\mathtt{size} = 1) * \varphi$ and $\blacklozenge^*_{\text{SL}} \varphi \overset{\text{def}}{=} \top * \varphi$. The semantics of these formulae is related to the analogous operators of ALT as follows:

$$(s,h) \models \blacklozenge_{\text{SL}} \varphi \iff \exists h_1, h_2 \text{ s.t. } h_1 \perp h_2,\ h_1 + h_2 = h,\ |\mathrm{dom}(h_1)| = 1 \text{ and } (s,h_2) \models \varphi.$$
$$(s,h) \models \blacklozenge^*_{\text{SL}} \varphi \iff \exists h_1, h_2 \text{ s.t. } h_1 \perp h_2,\ h_1 + h_2 = h \text{ and } (s,h_2) \models \varphi.$$

In order to perform the reduction from SAT(ALT) to SAT(1SL($*$, alloc, $\hookrightarrow^+$)), we fix a variable $\mathtt{x} \in \mathsf{VAR}$ that is syntactically different from u and that plays the role of the target node. Then, the translation $\tau_{\mathtt{x}}(\varphi)$ of a formula $\varphi$ in ALT is straightforward:

$$\begin{array}{lll} \tau_{\mathtt{x}}(\mathrm{T}) \overset{\text{def}}{=} \mathtt{u} \hookrightarrow^{+} \mathtt{x}. & \tau_{\mathtt{x}}(\blacklozenge \varphi) \overset{\text{def}}{=} \blacklozenge_{\text{SL}} \tau_{\mathtt{x}}(\varphi). & \tau_{\mathtt{x}}(\top) \overset{\text{def}}{=} \top. \\ \tau_{\mathtt{x}}(\mathrm{G}) \overset{\text{def}}{=} \mathtt{alloc}(\mathtt{u}) \wedge \neg \tau_{\mathtt{x}}(\mathrm{T}). & \tau_{\mathtt{x}}(\blacklozenge^* \varphi) \overset{\text{def}}{=} \blacklozenge^*_{\text{SL}} \tau_{\mathtt{x}}(\varphi). & \tau_{\mathtt{x}}(\neg\varphi) \overset{\text{def}}{=} \neg \tau_{\mathtt{x}}(\varphi). \\ \tau_{\mathtt{x}}(\langle \mathrm{U} \rangle \varphi) \overset{\text{def}}{=} \exists \mathtt{u}\ \tau_{\mathtt{x}}(\varphi). & \tau_{\mathtt{x}}(\varphi \wedge \psi) \overset{\text{def}}{=} \tau_{\mathtt{x}}(\varphi) \wedge \tau_{\mathtt{x}}(\psi). & \end{array}$$

Given a pointed forest $(\mathcal{F}, \mathsf{t}, \mathsf{n})$ and a store $s$ such that $s(\mathtt{x}) = \mathsf{t}$ and $s(\mathtt{u}) = \mathsf{n}$, by structural induction on $\varphi$ we can easily show that $(\mathcal{F}, \mathsf{t}, \mathsf{n}) \models \varphi \Leftrightarrow (s, \mathcal{F}) \models \tau_{\mathtt{x}}(\varphi)$. This, together with the fact that $\forall \mathtt{u}\ \neg(\mathtt{u} \hookrightarrow^{+} \mathtt{u})$ characterises the class of acyclic heaps (which correspond to the forests of ALT), directly implies the following result.

**Lemma 8.** *Let* $\mathtt{x} \in \mathsf{VAR} \setminus \{\mathtt{u}\}$. $\varphi$ *in* ALT *and* $\tau_{\mathtt{x}}(\varphi) \wedge \forall \mathtt{u}\ \neg(\mathtt{u} \hookrightarrow^{+} \mathtt{u})$ *are equisatisfiable.*

This lemma reproves that both 1SL($*$, alloc, $\hookrightarrow^+$) and first order separation logic with two quantified variables (denoted as 2SL($*$)) admit a TOWER-hard satisfiability problem. 2SL($*$), as introduced in [17], can be defined from 1SL($*$, alloc, $\hookrightarrow^+$) by removing alloc and $\hookrightarrow^+$ from the syntax and allowing a second variable, different from u, to be quantified. However, in [17] the authors show that both alloc and $\hookrightarrow^+$ are expressible in 2SL($*$), and with some very minor modification to their formulae we can show that both predicates are definable using $\blacklozenge$ and $\blacklozenge^*$ instead of $*$ and emp. Moreover, these logics are in TOWER by Rabin's Theorem [36], leading to the TOWER-completeness of SAT(ALT).

**Theorem 1.** SAT(2SL($*$)) *and* SAT(1SL($*$, alloc, $\hookrightarrow^+$)) *are* TOWER-*complete even when* emp *and* $*$ *are replaced with* $\blacklozenge_{\text{SL}}$ *and* $\blacklozenge^*_{\text{SL}}$. SAT(ALT) *is* TOWER-*complete.*

## 4.2 From ALT to Quantified Computation Tree Logic

We now consider Computation Tree Logic (CTL), a well-known logic for branching time model checking [14,13]. Among its extensions, in [5,22,28] the addition of propositional quantification is considered. The satisfiability problem of the resulting logic is undecidable on Kripke structures, and TOWER-complete on trees [28]. In [5], the authors show that the problem is TOWER-hard even when considering just one operator among *exists-next* $\mathsf{EX}$ or *exists-finally* $\mathsf{EF}$ (the definitions are below). Here, we reprove the result for $\mathsf{EF}$ by first tackling the TOWER-hardness of the logic with the *exists-until* $\mathsf{E}(\varphi \mathbin{\mathsf{U}} \psi)$, and then show that this operator can be defined using $\mathsf{EF}$. Differently from [5] and thanks to the properties of ALT, our reduction does not imbricate until operators, showing that this extension of CTL remains TOWER-hard even when $\mathsf{E}(\varphi \mathbin{\mathsf{U}} \psi)$ is restricted so that $\varphi$ and $\psi$ are Boolean combinations of propositional symbols.

Let us first recall the standard definition of Kripke structure [27]. Let $\mathsf{AP} \stackrel{\text{def}}{=} \{p, q, \cdots\}$ be a countable set of *propositional symbols*. A *Kripke structure* is a triple $(\mathcal{W}, \mathcal{R}, \mathcal{V})$ where $\mathcal{W}$ is a countable set of *worlds*, $\mathcal{R} \subseteq \mathcal{W} \times \mathcal{W}$ is a left-total *accessibility relation* (left-total means that for each world $\mathsf{w} \in \mathcal{W}$ there is $\mathsf{w}' \in \mathcal{W}$ s.t. $(\mathsf{w}, \mathsf{w}') \in \mathcal{R}$) and $\mathcal{V} : \mathsf{AP} \to 2^{\mathcal{W}}$ is a *labelling function*. We define $\mathcal{R}(\mathsf{w}) \stackrel{\text{def}}{=} \{\mathsf{w}' \in \mathcal{W} \mid (\mathsf{w}, \mathsf{w}') \in \mathcal{R}\}$ as the set of worlds accessible from $\mathsf{w} \in \mathcal{W}$. Let $\mathcal{R} \subseteq \mathcal{W} \times \mathcal{W}$ be an arbitrary relation on worlds (not necessarily left-total). A *path* $\pi$ starting in $\mathsf{w}$ is a sequence of worlds $(\mathsf{w}_0, \mathsf{w}_1, \cdots)$ such that $\mathsf{w}_0 = \mathsf{w}$ and $(\mathsf{w}_i, \mathsf{w}_{i+1}) \in \mathcal{R}$ for every two successive elements $\mathsf{w}_i, \mathsf{w}_{i+1}$ of the sequence. The path $\pi$ is said to be *maximal* whenever it is not a strict prefix of any other path. We denote with $\Pi_{\mathcal{R}}(\mathsf{w})$ the set of *maximal paths* starting in $\mathsf{w}$. If $\mathcal{R}$ is left-total then $\Pi_{\mathcal{R}}(\mathsf{w})$ is the set of all infinite paths starting in $\mathsf{w}$. Lastly, $\mathcal{R}^*(\mathsf{w})$ denotes the set of worlds reachable from $\mathsf{w}$, i.e. those worlds belonging to a path in $\Pi_{\mathcal{R}}(\mathsf{w})$.

We consider Quantified Computational Tree Logic interpreted under tree semantics ($\mathsf{QCTL}^T$) and refer the reader to [28] for a complete description of the logic. The formulae of $\mathsf{QCTL}^T$ are built from the following grammar:

$$\varphi := \top \mid \varphi \land \varphi \mid \neg\varphi \mid p \mid \mathsf{EX}\,\varphi \mid \mathsf{E}(\varphi \mathbin{\mathsf{U}} \varphi) \mid \mathsf{A}(\varphi \mathbin{\mathsf{U}} \varphi) \mid \exists p\, \varphi$$

where $p \in \mathsf{AP}$. All temporal modalities of $\mathsf{QCTL}^T$ are from CTL: $\mathsf{EX}$ is the *exists-next* modality, $\mathsf{E}(\varphi \mathbin{\mathsf{U}} \psi)$ is the *exists-until* modality and $\mathsf{A}(\varphi \mathbin{\mathsf{U}} \psi)$ is the *all-until* modality.

$\mathsf{QCTL}^T$ is interpreted on Kripke trees. Formally, a Kripke structure $(\mathcal{W}, \mathcal{R}, \mathcal{V})$ is a *(finitely-branching) Kripke tree* if (I) $\mathcal{R}^{-1}$ is functional and acyclic, (II) for every world $\mathsf{w} \in \mathcal{W}$, $\mathcal{R}(\mathsf{w})$ is finite and (III) it has a *root*, i.e. $\mathcal{R}^*(\mathsf{r}) = \mathcal{W}$ for some $\mathsf{r} \in \mathcal{W}$. Given $\mathsf{w} \in \mathcal{W}$, the worlds in $\mathcal{R}^*(\mathsf{w}) \setminus \{\mathsf{w}\}$ are said to be *descendants* of $\mathsf{w}$. As Kripke structures are left-total, Kripke trees can be seen as finitely-branching infinite trees. This leads to $\mathsf{SAT}(\mathsf{QCTL}^T)$ being in TOWER by reduction to MSO on trees [28]. Let $\mathcal{K} = (\mathcal{W}, \mathcal{R}, \mathcal{V})$ be a Kripke tree and $\mathsf{w} \in \mathcal{W}$. The satisfaction relation $\models$ of $\mathsf{QCTL}^T$ is defined as:

$(\mathcal{K}, \mathsf{w}) \models p \quad \stackrel{\text{def}}{\Leftrightarrow} \quad \mathsf{w} \in \mathcal{V}(p)$.

$(\mathcal{K}, \mathsf{w}) \models \mathsf{EX}\,\varphi \quad \stackrel{\text{def}}{\Leftrightarrow} \quad \exists \mathsf{w}' \in \mathcal{R}(\mathsf{w})$ s.t. $(\mathcal{K}, \mathsf{w}') \models \varphi$.

$(\mathcal{K}, \mathsf{w}) \models \mathsf{E}(\varphi \mathbin{\mathsf{U}} \psi) \quad \stackrel{\text{def}}{\Leftrightarrow} \quad$ there are $(\mathsf{w}_0, \mathsf{w}_1, \cdots) \in \Pi_{\mathcal{R}}(\mathsf{w})$ and $j \in \mathbb{N}$ such that $(\mathcal{K}, \mathsf{w}_j) \models \psi$ and for every $i < j$, $(\mathcal{K}, \mathsf{w}_i) \models \varphi$.

$(\mathcal{K}, \mathsf{w}) \models \mathsf{A}(\varphi \mathbin{\mathsf{U}} \psi) \quad \stackrel{\text{def}}{\Leftrightarrow} \quad$ for all $(\mathsf{w}_0, \mathsf{w}_1, \cdots) \in \Pi_{\mathcal{R}}(\mathsf{w})$, $\exists j \in \mathbb{N}$ such that $(\mathcal{K}, \mathsf{w}_j) \models \psi$ and for every $i < j$, $(\mathcal{K}, \mathsf{w}_i) \models \varphi$.

$(\mathcal{K}, \mathsf{w}) \models \exists p\, \varphi \quad \stackrel{\text{def}}{\Leftrightarrow} \quad$ there is $\mathcal{W}' \subseteq \mathcal{W}$ such that $(\mathcal{W}, \mathcal{R}, \mathcal{V}[p \leftarrow \mathcal{W}']) \models \varphi$, where, similarly to the store update $s[\mathtt{u} \leftarrow \ell']$ of the previous section, $\mathcal{V}[p \leftarrow \mathcal{W}']$ stands for the function obtained from $\mathcal{V}$ by updating the evaluation of $p$ from $\mathcal{V}(p)$ to $\mathcal{W}'$.

The formula $\exists p\ \varphi$ requires to update the satisfaction of $p$ in a way such that $\varphi$ is satisfied. This should already give a good clue on how to reduce ALT to $\mathsf{QCTL}^{T}$: we represent the nodes of a forest as the set of worlds satisfying a propositional symbol $D$. Then, for instance, the repeated sabotage operator $\blacklozenge^*$ is encoded by using an existential $\exists E$ that changes the evaluation of a propositional symbol $E$ so that it only holds in worlds where $D$ holds. In this way, the set of worlds satisfying $E$ represents a subforest of the original one. The universal quantification $\forall$ and the connectives $\Rightarrow$ and $\vee$ are defined as usual. So are the classical temporal operators from [14], *exists-finally* $\mathsf{EF}\,\varphi \stackrel{\text{def}}{=} \mathsf{E}(\top\ \mathsf{U}\ \varphi)$, *all-generally* $\mathsf{AG}\,\varphi \stackrel{\text{def}}{=} \neg\mathsf{EF}\,\neg\varphi$, *all-finally* $\mathsf{AF}\,\varphi \stackrel{\text{def}}{=} \mathsf{A}(\top\ \mathsf{U}\ \varphi)$, *exists-generally* $\mathsf{EG}\,\varphi \stackrel{\text{def}}{=} \neg\mathsf{AF}\,\neg\varphi$, and *exists-strong-release* $\mathsf{E}(\varphi\ \mathsf{M}\ \psi) \stackrel{\text{def}}{=} \mathsf{E}(\varphi\ \mathsf{U}\ \varphi \wedge \psi)$.

We now work towards a formal encoding of a pointed forest $(\mathcal{F}, \mathsf{t}, \mathsf{n})$ into a *pointed model* $(\mathcal{K}, \mathsf{w})$, where $\mathcal{K} = (\mathcal{W}, \mathcal{R}, \mathcal{V})$ is a Kripke tree and $\mathsf{w}$ is one of its worlds. We use $\mathsf{w}$ to play the role of the target node $\mathsf{t}$. To encode the forest $\mathcal{F}$ and the current evaluation node $\mathsf{n}$ we use the worlds appearing in $\mathcal{R}^*(\mathsf{w})$ and three propositional symbols: $D$, $end$ and $n$. The intended use of $D$ is to state which elements of $\mathcal{R}^*(\mathsf{w})$ encode nodes in $\mathrm{dom}(\mathcal{F})$. We need to be careful here, as $\mathcal{R}^*(\mathsf{w})$ is an infinite set whereas $\mathrm{dom}(\mathcal{F})$ is finite. We use the propositional symbol $end$ to solve this inconsistency: we constraint $\mathcal{K}$ to satisfy the formula $\mathsf{AF}(end)$ stating that every maximal path $(\mathsf{w}_0, \mathsf{w}_1, \cdots) \in \Pi_{\mathcal{R}}(\mathsf{w})$ has a finite prefix $(\mathsf{w}_0, \cdots, \mathsf{w}_{j-1})$ $(j \in \mathbb{N})$ of worlds not satisfying $end$, whereas $\mathsf{w}_j \in \mathcal{V}(end)$. Then, a world in $\mathcal{W}$ encodes an element in $\mathrm{dom}(\mathcal{F})$ whenever it satisfies $D$ and it belongs to one of these prefixes. We use the propositional symbol $n$ to encode the current evaluation node. During the translation we require $n$ to be satisfied by exactly one descendant of $\mathsf{w}$, so that the modality $\langle \mathrm{U} \rangle$ roughly becomes a quantification over $n$. From [28], checking whether a formula $\varphi$ holds in exactly one descendant of $\mathsf{w}$ can be done with the formula $\mathtt{uniq}(\varphi) \stackrel{\text{def}}{=} \mathsf{EF}(\varphi) \wedge \forall p\ (\mathsf{EF}(\varphi \wedge p) \Rightarrow \mathsf{AG}(\varphi \Rightarrow p))$ where $p \in \mathsf{AP}$ does not appear in $\varphi$. For technical reasons, we treat in a similar way the world $\mathsf{w}$, which encodes the target node, and require it to be the only world (among the ones in $\mathcal{R}^*(\mathsf{w})$) satisfying the auxiliary propositional symbol $t$. Lastly, we use an additional propositional symbol $E$ in order to encode subforests and deal with the encoding of $\blacklozenge$ and $\blacklozenge^*$ (as stated above).

We now formalise the encoding. For the remaining of this section, we fix a tuple $X \stackrel{\text{def}}{=} (end, n, t)$ of three different propositional symbols. Let $D$ be an additional symbol not in $X$, and let $(\mathcal{F}, \mathsf{t}, \mathsf{n})$ be a pointed forest s.t. $\mathsf{t} \notin \mathrm{dom}(\mathcal{F})$ (by Lemma 4(1) it is sufficient to consider this class of structures in order to decide satisfiability of a formula in ALT). A pointed model $(\mathcal{K} = (\mathcal{W}, \mathcal{R}, \mathcal{V}), \mathsf{w})$, is an $(X, D)$-*encoding* of $(\mathcal{F}, \mathsf{t}, \mathsf{n})$, or simply *encoding* when $(X, D)$ is clear from the context, if there is an injection $\mathfrak{f}: \mathcal{N} \to \mathcal{R}^*(\mathsf{w})$ s.t.

1. $\mathfrak{f}(\mathsf{t}) \stackrel{\text{def}}{=} \mathsf{w}$ is the only world in $\mathrm{ran}(\mathfrak{f}) \cap \mathcal{V}(t)$, and $\mathfrak{f}(\mathsf{n})$ is the only world in $\mathrm{ran}(\mathfrak{f}) \cap \mathcal{V}(n)$;
2. for every $\mathsf{n}' \in \mathrm{dom}(\mathcal{F})$ it holds that $(\mathfrak{f}(\mathcal{F}(\mathsf{n}')), \mathfrak{f}(\mathsf{n}')) \in \mathcal{R}$;
3. for every infinite path $(\mathsf{w}_0, \mathsf{w}_1 \cdots) \in \Pi_{\mathcal{R}}(\mathsf{w})$ there is $i \geq 0$ s.t. $\mathsf{w}_i \in \mathcal{V}(end)$ and
   - $\forall j \in [0, i-1]$, $\mathsf{w}_j \notin \mathcal{V}(end)$ and $(\mathsf{w}_j \in \mathcal{V}(D) \Leftrightarrow \exists \mathsf{n}' \in \mathrm{dom}(\mathcal{F})\ \mathfrak{f}(\mathsf{n}') = \mathsf{w}_j)$;
   - for every $j \geq i$ and every node $\mathsf{n}' \in \mathrm{dom}(\mathcal{F})$, $\mathfrak{f}(\mathsf{n}') \neq \mathsf{w}_j$.

It is easy to show that such an encoding always exists. Informally, the first property states that $\mathsf{w}$ encodes $\mathsf{t}$ and is the only world in $\mathcal{R}^*(\mathsf{w})$ satisfying $t$. Similarly, the world $\mathfrak{f}(\mathsf{n})$ encoding $\mathsf{n}$ is the only world in $\mathcal{R}^*(\mathsf{w})$ that satisfies $n$. The second property states that the forest must be correctly encoded in the Kripke structure. In particular, notice that the parent relation of the finite forest is inverted so that it becomes the child relation in the

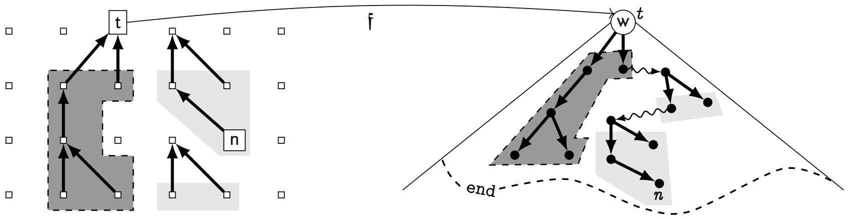

**Fig. 4.** A pointed forest (left) and one of its encoding as a finitely-branching Kripke tree (right).

Kripke structure (as shown in Figure 4). As $\mathfrak{f}$ is an injection, the encoding does not merge together trees that are disconnected in the forest. Lastly, the third property of $\mathfrak{f}$ states that the elements in $\mathrm{dom}(\mathcal{F})$ must be encoded by nodes in $\mathcal{R}^*(\mathsf{w})$ that precede every world satisfying $\mathit{end}$. Moreover, among all the descendants of $\mathsf{w}$ preceding $\mathit{end}$, the worlds encoding $\mathrm{dom}(\mathcal{F})$ are the only ones satisfying $D$. This implies that $\mathsf{w}$ does not satisfy $D$ (as $\mathsf{t} \notin \mathrm{dom}(\mathcal{F})$). Figure 4 shows a pointed forest and one of its possible encodings.

We now formalise the translation. Fix two different symbols $D, E$ not in $X$. In order to alternate between $D$ and $E$, we define $\overline{D} \stackrel{\text{def}}{=} E$ and $\overline{E} \stackrel{\text{def}}{=} D$. The translation $\tau_u(\varphi)$ of a formula $\varphi$ in ALT, implicitly parametrised by $X$ and where $u \in \{D, E\}$, is homomorphic for $\top$ and Boolean connectives (as in $\tau_X$, see Section 4.1), and otherwise it is defined as

$$\tau_u(\mathrm{T}) \stackrel{\text{def}}{=} \mathsf{E}(((u \vee t) \wedge \neg \mathit{end})\ \mathsf{M}\ (u \wedge n)). \qquad \tau_u(\langle \mathrm{U} \rangle\, \varphi) \stackrel{\text{def}}{=} \exists n\ (\mathtt{uniq}(n) \wedge \tau_u(\varphi)).$$
$$\tau_u(\mathrm{G}) \stackrel{\text{def}}{=} \mathsf{E}(\neg \mathit{end}\ \mathsf{M}\ (u \wedge n)) \wedge \neg\, \tau_u(\mathrm{T}). \qquad \tau_u(\blacklozenge^* \varphi) \stackrel{\text{def}}{=} \exists \overline{u}\ (\mathsf{AG}\,(\overline{u} \Rightarrow u) \wedge \tau_{\overline{u}}(\varphi)).$$
$$\tau_u(\blacklozenge\, \varphi) \stackrel{\text{def}}{=} \exists \overline{u}\ (\mathsf{AG}\,(\overline{u} \Rightarrow u) \wedge \mathtt{uniq}(u \wedge \neg \overline{u}) \wedge \mathsf{E}(\neg \mathit{end}\ \mathsf{M}\ (u \wedge \neg \overline{u})) \wedge \tau_{\overline{u}}(\varphi)).$$

Let $(\mathcal{F}, \mathsf{t}, \mathsf{n})$ be a pointed forest s.t. $\mathsf{t} \notin \mathrm{dom}(\mathcal{F})$ and let $((\mathcal{W}, \mathcal{R}, \mathcal{V}), \mathsf{w})$ be one of its $(X, u)$-encodings w.r.t. the injection $\mathfrak{f}$. For instance, $\tau_u(\mathrm{T})$ requires that there is a path $(\mathsf{w}, \mathsf{w}_1, \cdots, \mathsf{w}_j)$ starting in $\mathfrak{f}(\mathsf{t}) = \mathsf{w}$ and whose worlds do not satisfy $\mathit{end}$ and must satisfy $u$ or $t$. Moreover, the last world $\mathsf{w}_j$ must satisfy $u$ and $n$. From property (1) of the definition of $\mathfrak{f}$, the only element satisfying $t$ is $\mathsf{w}$, which does not satisfy $u$ (as $\mathsf{t} \notin \mathrm{dom}(\mathcal{F})$). Then, this path of worlds encodes a path in the pointed forest, from the current evaluation node $\mathsf{n}$ (which is encoded by the only world satisfying $n$) to the target node $\mathsf{t}$. The translation is shown correct (by structural induction on $\varphi$) for pointed forests that admit an encoding.

**Lemma 9.** *Let* $(\mathcal{F}, \mathsf{t}, \mathsf{n})$ *be a pointed forest s.t.* $\mathsf{t} \notin \mathrm{dom}(\mathcal{F})$*, and let* $(\mathcal{K}, \mathsf{w})$ *be a* $(X, u)$*-encoding of* $(\mathcal{F}, \mathsf{t}, \mathsf{n})$*. Given a formula* $\varphi$ *in* ALT*,* $(\mathcal{F}, \mathsf{t}, \mathsf{n}) \models \varphi$ *if and only if* $(\mathcal{K}, \mathsf{w}) \models \tau_u(\varphi)$*.*

Then, to conclude the reduction we just need to characterise the set of models encoding a pointed forest. The formula $\mathtt{enc} \stackrel{\text{def}}{=} \neg D \wedge t \wedge \mathtt{uniq}(t) \wedge \mathtt{uniq}(n) \wedge \mathsf{AF}(\mathit{end})$ does the job.

**Lemma 10.** $\varphi$ *in* ALT *and* $\mathtt{enc} \wedge \tau_D(\varphi)$ *in* $\mathsf{QCTL}^T$ *are equisatisfiable.*

We now take a closer look to the translation. Given a temporal modality $\mathcal{T}$ and $k \in \mathbb{N} \cup \{\omega\}$, $\mathsf{QCTL}^T(\mathcal{T}^k)$ denotes the fragment of $\mathsf{QCTL}^T$ restricted to formulae where the only temporal modality allowed is $\mathcal{T}$, which can be nested at most $k$ times ($\omega$ stands for an arbitrary number of imbrications). For instance, $\mathsf{QCTL}^T(\mathsf{EF}^k)$ denotes the set of formulae restricted to the operator $\mathsf{EF}$, which can be nested at most $k$ times. This fragment of $\mathsf{QCTL}^T$ is shown to be $k$-NExpTime-hard in [5], which directly leads to the Tower-hardness of $\mathsf{QCTL}^T(\mathsf{EF}^\omega)$ and $\mathsf{QCTL}^T(\mathsf{EU}^\omega)$. By analysing our translation it is easy to show that $\mathsf{QCTL}^T(\mathsf{EU}^0)$, i.e. $\mathsf{QCTL}^T$ restricted to the only modality $\mathsf{E}(\varphi \,\mathsf{U}\, \psi)$ where $\varphi$ and $\psi$ are Boolean combination of propositional symbols, and $\mathsf{QCTL}^T(\mathsf{EF}^1)$ are already TOWER-hard. First of all, the formula $\mathsf{E}(\varphi \,\mathsf{U}\, \psi)$ in $\mathsf{QCTL}^T(\mathsf{EU}^0)$ is equivalent to the following formula in $\mathsf{QCTL}^T(\mathsf{EF}^1)$: $\exists p\big(\mathsf{AG}\,(\neg\varphi \wedge \neg\psi \Rightarrow p) \wedge \mathsf{AG}\,(p \Rightarrow \mathsf{AG}\,p) \wedge \mathsf{EF}\,(\psi \wedge \neg p)\big)$, where $p$ does not appear in $\varphi$ or $\psi$. Then, we just need to prove the result for $\mathsf{QCTL}^T(\mathsf{EU}^0)$.

Clearly, the translation $\tau_u$ is defined so that the resulting formula is in $\mathsf{QCTL}^T(\mathsf{EU}^0)$. However, we need to deal with the occurrence of $\mathsf{AF}\,(\mathit{end})$ used inside the formula enc. Let us first consider the formula $\mathsf{AG}\,(\varphi \Rightarrow \mathsf{AG}\,\psi)$ which is satisfied by models where once $\varphi$ is found to hold in a certain world w, then $\psi$ is satisfied in every world of $\mathcal{R}^*(\mathsf{w})$. Despite not being in $\mathsf{QCTL}^T(\mathsf{EU}^0)$, the formula $\mathsf{AG}\,(\varphi \Rightarrow \mathsf{AG}\,\psi)$ is equivalent to the following formula: $\forall p \forall q\ \big(\mathtt{uniq}(p) \wedge \mathtt{uniq}(q) \wedge \mathsf{EF}\,(p \wedge \varphi) \wedge \mathsf{EF}\,(q \wedge \neg\psi) \Rightarrow \mathsf{E}(\neg p \,\mathsf{M}\, q)\big)$, where $p$ and $q$ do not appear in $\varphi$ or $\psi$. We then define a formula $\chi_{\mathsf{EG}}\,(\varphi)$ that only uses $\mathsf{EF}$ modalities and is equivalent to $\mathsf{EG}\,\varphi$, so that then $\neg\chi_{\mathsf{EG}}\,(\neg\varphi)$ is equivalent to $\mathsf{AF}\,\varphi$:

$$\begin{aligned}\chi_{\mathsf{EG}}\,(\varphi) \stackrel{\text{def}}{=} \exists p\big(&\neg p \wedge \mathsf{AG}\,(\neg\varphi \Rightarrow p) \wedge \mathsf{AG}\,(p \Rightarrow \mathsf{AG}\,p) \wedge \\ &\forall q\big(\mathtt{uniq}(q) \wedge \mathsf{EF}\,(q \wedge \neg p) \Rightarrow \mathsf{EF}\,(q \wedge \mathsf{EF}\,(\neg q \wedge \neg p))\big)\big)\end{aligned}$$

where $p$ does not appear in $\varphi$. This formula is expressible in $\mathsf{QCTL}^T(\mathsf{EU}^0)$, as every subformula that is not in this fragment is an instance of $\mathsf{AG}\,(\varphi \Rightarrow \mathsf{AG}\,\psi)$. Then, we conclude that $\mathsf{AF}\,(\mathit{end})$ is expressible in $\mathsf{QCTL}^T(\mathsf{EU}^0)$, leading to the following result.

**Theorem 2.** *The satisfiability problems of* $\mathsf{QCTL}^T(\mathsf{EU}^0)$ *and* $\mathsf{QCTL}^T(\mathsf{EF}^1)$ *are* TOWER*-c.*

### 4.3 From ALT to Modal Logic of Heaps and Modal Separation Logic

In [17] and later in [18] two families of logics are presented, respectively called *modal logic of heaps* (MLH) and *modal separation logic* (MSL). At their core, both logics can be seen as modal logics extended with separating connectives, hence mixing separation logic (Section 4.1) with temporal aspects as in quantified CTL (Section 4.2). As we already shown how ALT is captured by these two latter logics, it is natural to ask ourselves if the same holds for MLH and MSL. In this section, we show that this is indeed the case and, as for the previous two sections, ALT allows us to refine the analysis on these logics. Both MLH and MSL are interpreted on finite Kripke functions. A *finite Kripke function* is a Kripke structure $(\mathcal{W}, \mathcal{R}, \mathcal{V})$ (see Section 4.2 for its definition) where $\mathcal{W}$ is infinite and $\mathcal{R}$, instead of being left-total, is finite and weakly functional, i.e. $|\mathcal{R}| \in \mathbb{N}$ and for every $\mathsf{w}, \mathsf{w}', \mathsf{w}'' \in \mathcal{W}$, if $(\mathsf{w}, \mathsf{w}') \in \mathcal{R}$ and $(\mathsf{w}, \mathsf{w}'') \in \mathcal{R}$ then $\mathsf{w}' = \mathsf{w}''$. As $\mathcal{N}$ and $\mathcal{W}$ are both countably infinite sets, without loss of generality we assume $\mathcal{W} = \mathcal{N}$. Two Kripke structures $\mathcal{K}_1 = (\mathcal{W}, \mathcal{R}_1, \mathcal{V})$ and $\mathcal{K}_2 = (\mathcal{W}, \mathcal{R}_2, \mathcal{V})$ are disjoint if $\mathcal{R}_1 \cap \mathcal{R}_2 = \emptyset$. When this holds, $\mathcal{K}_1 + \mathcal{K}_2$ denotes the model $(\mathcal{W}, \mathcal{R}_1 \cup \mathcal{R}_2, \mathcal{V})$. To shorten the presentation, in the following diagram we introduce a language having the operators from MSL and MLH, and summarise known and new results on these logics (where $p \in \mathsf{AP}$):

MSL: TOWER-complete from [18]. MLH: TOWER-complete from [17].

$$\varphi := p \mid \langle\neq\rangle\,\varphi \mid \top \mid \varphi \wedge \varphi \mid \neg\varphi \mid \Diamond\varphi \mid \varphi * \psi \mid \langle \mathrm{U}\rangle\,\varphi \mid \Diamond^{-1}\varphi$$

TOWER-hard by reduction from SAT(ALT), shown here.

As defined below, $\Diamond$ is the standard alethic modality from modal logic, $\Diamond^{-1}$ is its converse modality, and $\langle\neq\rangle$ is the *elsewhere* modality that generalises the somewhere modality $\langle\mathrm{U}\rangle$ as $\langle\mathrm{U}\rangle\,\varphi = \varphi \vee \langle\neq\rangle\,\varphi$. For a *pointed model* $(\mathcal{K}, \mathsf{w})$, where $\mathcal{K} = (\mathcal{W}, \mathcal{R}, \mathcal{V})$ is a finite Kripke function and $\mathsf{w} \in \mathcal{W}$, the satisfaction relation $\models$ is defined as follows:

$$\begin{array}{lcl}
(\mathcal{K},\mathsf{w}) \models p & \overset{\text{def}}{\Leftrightarrow} & \mathsf{w} \in \mathcal{V}(p).\\
(\mathcal{K},\mathsf{w}) \models \Diamond\varphi & \overset{\text{def}}{\Leftrightarrow} & \text{there is } \mathsf{w}' \in \mathcal{R}(\mathsf{w}) \text{ such that } (\mathcal{K},\mathsf{w}) \models \varphi.\\
(\mathcal{K},\mathsf{w}) \models \Diamond^{-1}\varphi & \overset{\text{def}}{\Leftrightarrow} & \text{there is } \mathsf{w}' \in \mathcal{W} \text{ such that } \mathsf{w} \in \mathcal{R}(\mathsf{w}') \text{ and } (\mathcal{K},\mathsf{w}') \models \varphi.\\
(\mathcal{K},\mathsf{w}) \models \langle\neq\rangle\,\varphi & \overset{\text{def}}{\Leftrightarrow} & \text{there is } \mathsf{w}' \in \mathcal{W} \text{ such that } \mathsf{w}' \neq \mathsf{w} \text{ and } (\mathcal{K},\mathsf{w}') \models \varphi.\\
(\mathcal{K},\mathsf{w}) \models \varphi * \psi & \overset{\text{def}}{\Leftrightarrow} & (\mathcal{K}_1,\mathsf{w}) \models \varphi \text{ and } (\mathcal{K}_2,\mathsf{w}) \models \psi \text{ for some } \mathcal{K}_1, \mathcal{K}_2 \text{ s.t. } \mathcal{K}_1 + \mathcal{K}_2 = \mathcal{K}.
\end{array}$$

By looking at the diagram above, compared to the work in [18], ALT allows us to show that propositional symbols and the elsewhere modality can be removed from MSL without changing the complexity status of its satisfiability problem. Similarly, ALT allows us to refine the analysis on the complexity of SAT(MLH) by showing that the $\Diamond^{-1}$ modality is not needed in order to achieve non-elementary complexities.

Let $(\mathcal{F}, \mathsf{t}, \mathsf{n})$ be a pointed forest and let $(\mathcal{K}, \mathsf{w})$ be a pointed model where $\mathcal{K} = (\mathcal{W}, \mathcal{R}, \mathcal{V})$. For the reduction, we use $\mathsf{w}$ to encode the current node $\mathsf{n}$. Encoding $\mathsf{t}$ is not so immediate, as MLH does not have propositional symbols. A possible solution is to encode it as a self-loop, so that the formula $\mathtt{T}$ is translated to a query stating that $\mathsf{w}$ reaches the self-loop. As done in Section 4.1 we define the formula $\mathtt{size{=}1} \overset{\text{def}}{=} \langle\mathrm{U}\rangle \Diamond\top \wedge \neg(\langle\mathrm{U}\rangle \Diamond\top * \langle\mathrm{U}\rangle \Diamond\top)$, that is satisfied whenever $|\mathcal{R}|{=}1$. We also define the modalities $\blacklozenge$ and $\blacklozenge^*$ in MLH: $\blacklozenge_{\text{ML}}\,\varphi \overset{\text{def}}{=} (\mathtt{size{=}1}) * \varphi$ and $\blacklozenge^*_{\text{ML}}\varphi \overset{\text{def}}{=} \top * \varphi$. Lastly, we introduce the formula $\mathtt{selfloop} \overset{\text{def}}{=} \blacklozenge^*_{\text{ML}}(\Diamond\Diamond\top \wedge \neg\blacklozenge_{\text{ML}}\blacklozenge_{\text{ML}}\top)$ that is satisfied by $(\mathcal{K}, \mathsf{w})$ if $(\mathsf{w}, \mathsf{w}) \in \mathcal{R}$. Suppose for a moment that we are able to use this formula to characterise the class of of every finite Kripke function $(\mathcal{W}, \mathcal{R}, \mathcal{V})$ where there is exactly one cycle, and this cycle is a self-loop on a world $\mathsf{w}_\mathsf{t}$. Then, we use $\mathsf{w}_\mathsf{t}$ to encode the target node $\mathsf{t}$ of a finite forest $(\mathcal{F}, \mathsf{t}, \mathsf{n})$ while being careful that the $\blacklozenge$ and $\blacklozenge^*$ operators of ALT are translated in such a way that the self-loop on $\mathsf{w}_\mathsf{t}$ is preserved. Because of the specific treatment of $\mathsf{w}_\mathsf{t}$, it is convenient to assume that the current evaluation node $\mathsf{n}$ is encoded by a world different from $\mathsf{w}_\mathsf{t}$, which reflects on the translation of $\langle\mathrm{U}\rangle$. The admissibility of this assumption follows by Lemma 4.

We encode pointed forests as finite Kripke functions. Let $(\mathcal{F}, \mathsf{t}, \mathsf{n})$ be a pointed forest s.t. $\mathsf{t} \notin \mathrm{dom}(\mathcal{F})$ and $\mathsf{n} \neq \mathsf{t}$. A finite Kripke function $((\mathcal{N}, \mathcal{R}, \mathcal{V}), \mathsf{n})$ (recall, $\mathcal{W} = \mathcal{N}$) is an *encoding* of $(\mathcal{F}, \mathsf{t}, \mathsf{n})$ iff for every $\mathsf{n}', \mathsf{n}'' \in \mathcal{N}$ we have $(\mathsf{n}', \mathsf{n}'') \in \mathcal{R} \Leftrightarrow (\mathcal{F}(\mathsf{n}') = \mathsf{n}''$ or $\mathsf{n}' = \mathsf{n}'' = \mathsf{t})$. Notice how $\mathcal{R}$ is essentially defined from $\mathcal{F}$ by adding the self-loop $(\mathsf{t}, \mathsf{t})$. The translation $\tau(\varphi)$ in MLH of a formula $\varphi$ in ALT is homomorphic for $\top$ and Boolean connectives (as is the case for $\tau_\mathtt{x}$ in Section 4.1), and otherwise it is defined as

$$\begin{array}{llll}
\tau(\mathtt{T}) & \overset{\text{def}}{=} \blacklozenge^*_{\text{ML}}\big(\Diamond\top \wedge [\mathrm{U}](\Diamond\top \Rightarrow \Diamond\Diamond\top)\big). & \tau(\blacklozenge\,\varphi) & \overset{\text{def}}{=} \blacklozenge_{\text{ML}}(\tau(\varphi) \wedge \langle\mathrm{U}\rangle\,\mathtt{selfloop}).\\
\tau(\mathtt{G}) & \overset{\text{def}}{=} \Diamond\top \wedge \neg\,\tau(\mathtt{T}). & \tau(\blacklozenge^*\varphi) & \overset{\text{def}}{=} \blacklozenge^*_{\text{ML}}(\tau(\varphi) \wedge \langle\mathrm{U}\rangle\,\mathtt{selfloop}).\\
\tau(\langle\mathrm{U}\rangle\,\varphi) & \overset{\text{def}}{=} \langle\mathrm{U}\rangle(\neg\mathtt{selfloop} \wedge \tau(\varphi)). & &
\end{array}$$

We highlight two points of this translation. First, $\tau(\mathtt{T})$ essentially asks to find a submodel where every path reaches the self-loop and the current evaluation node is in one of these paths. Second, notice how the translation of $\blacklozenge$ and $\blacklozenge^*$ checks that the model is updated so that the self-loop is not lost, as required by our encoding. It should be noted that this requirement cannot be met if we were translating the definition of ALT from [31], featuring the $*$ operator. Indeed, by partitioning the model into two pieces, this operator removes the self-loop from one of the two parts, breaking our encoding. The following lemma (proved by structural induction on $\varphi$) shows the correctness of our translation.

**Lemma 11.** *Let* $(\mathcal{F}, \mathsf{t}, \mathsf{n})$ *be a pointed model s.t.* $\mathsf{n} \neq \mathsf{t}$ *and* $\mathsf{t} \notin \mathrm{dom}(\mathcal{F})$. *Let* $(\mathcal{K}, \mathsf{n})$ *be an encoding of* $(\mathcal{F}, \mathsf{t}, \mathsf{n})$. *Given a formula* $\varphi$ *in* ALT, $(\mathcal{F}, \mathsf{t}, \mathsf{n}) \models \varphi$ *iff* $(\mathcal{K}, \mathsf{n}) \models \tau(\varphi)$.

To conclude the reduction we show that we can characterise the class of models encoding pointed forests, i.e. the finite Kripke functions with exactly one cycle, which is a self-loop. We first define the formula $\mathtt{hascycl} \stackrel{\text{def}}{=} \blacklozenge^{*}_{\text{ML}}\big(\langle \mathrm{U}\rangle \Diamond\top \wedge [\mathrm{U}](\Diamond\top \Rightarrow \Diamond\Diamond\top)\big)$ that checks if a finite Kripke function has at least one cycle. Then, the desired property can be simply defined by stating that there is a self-loop which, whenever removed, leads to an acyclic submodel: $\mathtt{1selfloop} \stackrel{\text{def}}{=} \langle \mathrm{U}\rangle \big(\mathtt{selfloop} \wedge \neg \blacklozenge_{\text{ML}}(\Box\bot \wedge \mathtt{hascycl})\big)$.

**Lemma 12.** *Every formula* $\varphi$ *in* ALT *is equisatisfiable with* $\tau(\varphi) \wedge \mathtt{1selfloop}$.

For the proof of Lemma 12, both Lemma 4(1) and (2) are used in order to restrict ourselves to pointed forest $(\mathcal{F}, \mathsf{t}, \mathsf{n})$ s.t. $\mathsf{n} \neq \mathsf{t}$ and $\mathsf{t} \notin \mathrm{dom}(\mathcal{F})$. Then, we apply Lemma 11.

**Theorem 3.** *The fragment of MLH and MSL with Boolean operators,* $\Diamond$ *and* $\langle \mathrm{U}\rangle$ *modalities, and* $*$ *(alternatively,* $\blacklozenge_{\text{ML}}$ *and* $\blacklozenge^{*}_{\text{ML}}$*) has a* TOWER*-complete satisfiability problem.*

## 5 Conclusions

We studied an *Auxiliary Logic on Trees* (ALT), a quite simple formalism that admits a TOWER-complete satisfiability problem. ALT is shown to be easily captured by various non-elementary logics: first-order separation logic, quantified CTL, modal logic of heaps and modal separation logic. Through ALT, we were not only able to connect these logics, but also to refine their analysis and find strict fragments that are still TOWER-hard. Most importantly, with ALT we hope to have shown a set of simple and concrete properties, centred around reachability and submodel reasoning, that when put together lead to logics having a non-elementary satisfiability problem.

This work leaves a few questions open. First, the fragments of ALT where $\blacklozenge$ or $\blacklozenge^{*}$ are removed from the logic have not being studied yet. The logic without $\blacklozenge^{*}$ is of particular interests, as it is connected with the sabotage logics from [4]. Second, the analysis done on first-order separation logic and on modal logic of heaps (Sections 4.1 and 4.3) reveals that the complexity of these logics does not change when the $*$ operator and the emp predicate are replaced with the less general operators $\blacklozenge$ and $\blacklozenge^{*}$. We find this point interesting, as from an overview of the literature, it seems that this result also holds for the separation logics considered in [9,17,19,30,31]. Moreover, for the logics whose expressiveness is known, i.e. the ones in [19,30], it seems that also the expressive power remains unchanged. However, we struggle to see how to uniformly express the operator $*$ with $\blacklozenge$ and $\blacklozenge^{*}$, as the resulting logics reason on the model in a different way (as as shown in Section 2). Lastly, this work illustrates the potential of ALT as a tool for proving the TOWER-hardness of logics interpreted on tree-like structures. As the operators of our logic are simple, we hope ALT to be useful to study logics with unknown complexities.

**Acknowlegements.** I would like to thank S. Demri and E. Lozes for their feedback.
